# Supplementary material for: scRNA-seq of gastric tumor shows complex intercellular interaction with an alternative T cell exhaustion trajectory
Source: Nat Commun. 2022 Aug 23;13:4943. doi: 10.1038/s41467-022-32627-z (PMC9399107; doi:10.1038/s41467-022-32627-z)

Supplementary information

Supplementary Methods

Supplementary Table

Supplementary Table 1 lists the signature genes of different epithelial cell types.
Supplementary Table 2 lists the primers used in primer sequences used in this paper.

Supplementary Figure

Supplementary Figure 1 is related to Figure 1.
Supplementary Figure 2 is related to Figure 2.
Supplementary Figure 3 and 4 are related to Figure 3.
Supplementary Figure 5 is related to Figure 4.
Supplementary Figure 6 and 7 are related to Figure 5.
Supplementary Figure 8 is related to Figure 6.
Supplementary Figure 9 is related to Figure 6 and 7.
Supplementary Figure 10 and 11 are related to Figure 8.
Supplementary Figure 12 is about subsets of NK, NKT, γδ T, and B cells.
Supplementary Figure 13 is about the evaluation of patient effects.
Supplementary Figure 14 is about the evaluation of cell quality difference.
Supplementary Figure 15 is about the evaluation of the robustness and necessity of T-cell clusters.
Supplementary Figure 16 is about the results of the whole-exome sequencing (WES) dataset.
Supplementary Figure 17 and 18 are the uncropped Western blots.

Supplementary Note

Supplementary Note 1 is the evaluation of batch effects
Supplementary Note 2 is the evaluation of the robustness and necessity of T-cell clusters.
Supplementary Note 3 is a case study of GC08 with abnormal Wnt signaling

Supplementary Data

Supplementary Data 1 contains the clinical characteristics of GC patients and sequencing information.
Supplementary Data 2 contains the conventional markers of each cell type used to identify doublets and the number of excluded cells for each cluster.
Supplementary Data 3 contains lists of differentially expressed genes (DEGs) in all cell clusters.
Supplementary Data 4 contains the list of common genes expressed by Tex and Treg and the list of common genes expressed by Tc17 and Th17.
Supplementary Data 5 contains the matrix of VDJ gene usages for each T cell cluster.
Supplementary Data 6 contains lists of ligand-receptor interaction pairs, related to Figure 8.
Supplementary Data 7 contains information on antibodies used for multiplex fluorescent immunohistochemistry.
Supplementary Data 8 contains the malignant gene set and the non-malignant gene set.

Supplementary Methods

Bulk RNA Sequencing and Whole Exome Sequencing (WES) of human frozen samples

Genomic DNA of blood and tissue samples were extracted using Monarch Genomic DNA Purification Kit (NEB, Cat: T3010L) and exome libraries were constructed using SureSelectXT Human All Exon V6 kit (Aligent, Cat: 5190-8864). RNA of tissue samples was extracted using the Monarch Total RNA Miniprep Kit (NEB, Cat: T2010S). After quality analysis, mRNA enrichment was carried out with NEBNext Poly(A) mRNA Magnetic Isolation Module kit (NEB, Cat: E7490L) and bulk RNA-seq libraries were constructed using NEBNext Ultra RNA Library Prep Kit for Illumina (NEB, Cat: E7530L). All experimental procedures followed manufacturer's specification. All libraries were sequenced on illumina Hiseq Xten or Novaseq with 150 bp paired-end reads.

Cell culture and TFs overexpression

The coding sequences of CDX2, HOXA13-flag tag, NR1I2-flag tag, TFEC and NR1H3 were amplified from cDNA in scRNA-seq experiment and were cloned into lentivirus vectors (pHAGE). Empty pHAGE vector was used as negative control. Lentivirus was packaged using packing and envelope plasmids psPAX2 and pMD2.G in LentiX cells as previously described [1]. THP-1 monocyte cells were cultured in RPMI 1640 medium containing 10% FBS, 1% penicillin/streptomycin (p/s), 12.5 mM HEPES (Thermo Fisher, Cat: 15630080) and 0.05 mM β-mercaptoethanol (Sigma, Cat: M3148) at 37˚C in 5% CO2. Nine gastric cancer cell lines, including SNU-16, KATO III, MKN-45, SNU-1, AGS, SGC-7901, HGC-27, MKN-28 and MKN-7, were cultured in RPMI 1640 medium containing 10% FBS, 1% p/s. THP-1, SGC-7901, HGC-27 and MKN-28 cells were transduced with concentrated virus, centrifuged at 1200 rpm for 100 min at 32°C, and replaced with fresh medium 12 hours later. Two days later, green fluorescent protein (GFP) positive cells were sorted for expansion using FACSAria III. Total cell protein extracts of transduced cells were made in

RIPA (Beyotime, Cat: P0013B), and were subjected to western blotting with anti-CDX2 (CST, Cat: 12306S; 1:1000), anti-TFEC (abcam, Cat: ab185226; 1:1000), anti-NR1H3 (abcam, Cat ab176323; 1:1000), and anit-Flag tag (CST, Cat: 14793S; 1:1000) antibody for validating the overexpression of target TFs. Primers used in this paper were listed in Supplementary Table 2.

Preparation of conditioned medium

Gastric cancer-associated fibroblasts (CAFs) were purchased from Zhejiang Meisen Cell Technology Co., Ltd, and were cultured in a primary cell culture medium (purchased from Zhejiang Meisen Cell Technology Co., Ltd, Hangzhou, China). When CAFs reached to a confluency of 80%, cell supernatants were discarded and washed twice with PBS, then fresh RPMI 1640 medium containing 10% FBS, 1% p/s were added to continue culturing. After 48 h, cell supernatants were collected and clarified by centrifugation, then were stored in -80℃ after mixed well with equal volume of fresh RPMI 1640 medium containing 20% FBS, 1% p/s for downstream experiments as conditioned medium.

CCK-8 assay

CCK-8 assay was carried out to evaluate the effect of conditioned mediums on gastric cancer cell proliferation. Briefly, 1.5 x 10^3 (MKN-28, AGS, SGC7901, HGC27, SNU-1) or 3 x 10^3 (MKN-7) cells were suspended in 100 μL conditioned medium or RPMI 1640 medium containing 10% FBS, 1% p/s in 96-well plates with six replicates. At 36 h, 100ul fresh corresponding mediums were added to continue culturing for another 36 h. After incubation, cell supernatants were discarded, fresh RPMI 1640 medium containing 10% FBS, 1% p/s and 10ul CCK-8 (Beyotime, Cat: C0039) were added into each well, and incubated for 180min. The absorbance at 450 nm was measured on a microplate reader (BioTek).

Stimulation in THP-1 cells

To assess the effect of TFEC and NR1H3 on macrophage state, TFEC and NR1H3 transduced THP-1 cells were seeded at the density of 1 x 10^5 cells in 48-well flat bottoms and were induced toward macrophages by 100 nM phorbol 12-myristate 13-acetate (PMA, Sigma, Cat: P8139) for 24 h. Then, cells were stimulated with either 100 ng/ml TLR agonist Pam3CSK4 (Invivogen, Cat: tlrl-pms) or 20 ng/ml lipopolysaccharide (LPS) (Beyotime, Cat: S1732) plus 20 ng/ml γ-interferon (IFNγ) (Beyotime, Cat: P5664) for 48 hours.

To assess the effect of cytokines secreting by gastric CAFs on macrophage polarization. THP-1 cells were seeded at the density of 2 x 10^5 cells in the lower chamber of transwell chamber with 8-μm pores (Corning, Cat: 3470) and were induced toward macrophages by 100 nM PMA for 24 h. In one group, 1 x 10^5 CAFs were seeded in the upper chamber. In the other group, conditioned medium of CAFs were directedly added into the lower chamber. The macrophages in these two groups were induced for 24, 48, 60 and 72 h with three replicates. At 48h, fresh mediums were added. After incubation, total RNA was extracted with TRIzol (Thermo, Cat: 10296028) for RNA-seq library construction by modified Smart-seq2 protocol. Briefly, total RNA was treated with DNase I (Thermo, Cat: 18068015) at 20°C for 10 min for DNA digestion with heat inactivation at 65°C for 10 min. 500ng RNA was used for reverse transcription by SuperScript ™ II (Thermo Fisher, Cat: 18064014). The cDNA was amplified using 8 PCR enrichment cycles before quantification and dual-index barcoding with the TruePrep DNA Library Prep Kit V2 (Vazyme, Cat: TD501) for Illumina. Libraries were sequenced on Illumina Novaseq with 150 bp paired-end reads.

RNA isolation and quantitative PCR analysis

Total RNA was extracted with TRIzol and was reverse-transcribed with RevertAid First Strand cDNA Synthesis Kit (Thermo Scientific, Cat: K1622) according to the manufacturer's instructions. Real-time PCR was performed with Power SYBR Green PCR Master Mix (Genestar, Cat: A311) and amplified using the ABI 7500 Real-Time PCR system (Applied Biosystems). Results were normalized to GAPDH and quantification was carried out using the $2^{-\Delta\Delta Ct}$ method. Melting curves were confirmed to ensure amplification of a single product.

Supplementary Table 1

Cell types	Signature genes
chief cell	*PGA4, PGA3, LIPF*
Basal gland mucous cell (GMC) or mucous neck cell	*MUC6, FUT9*
pit mucous cell (PMC) (Surface mucous cell)	*MUC5AC, TFF1, TFF2, GKN1*
parietal cell	*ATP4A, ATP4B, GIF*
goblet cell	*MUC2, ATOH1, TFF3, SPINK4, CLCA1, FCGBP*
enterocyte	*FABP1, VIL1, CDX1, CDX2, REG4, KRT20*

Supplementary Table 2

Primers name	Sequence
CDX2_cDNA Forward	CATTTCAGGTGTCGTGAAGCATGTACGTGAGCTACCTCCTGGAC
CDX2_cDNA Reverse	TCATCCTTGTAATCGATCCTCTGGGTGACGGTGGGGTTTAGC
NR1H3_cDNA Foward	CATTTCAGGTGTCGTGAAGCATGTCCTTGTGGCTGGGGGC
NR1H3_cDNA Reverse	AGGGATCCTCTAGACTCGAGTCATTCGTGCACATCCCAGATCTC
TFEC_cDNA Foward	CATTTCAGGTGTCGTGAAGCATGACCCTTGATCATCAGATCATCA
TFEC_cDNA Reverse	AGGGATCCTCTAGACTCGAGTCATAATTCATCACCATCATCTGAGCT
HOXA13_cDNA Forward	CATTTCAGGTGTCGTGAAGCATGACAGCCTCCGTGCTCCTC
HOXA13_cDNA Reverse	TCATCCTTGTAATCGATCCTTCATCAACAAACTGAAAACCACTAGT
NR1I2_cDNA Forward	CATTTCAGGTGTCGTGAAGCATGGAGGTGAGACCCAAAGAAAGCT
NR1I2_cDNA Reverse	TCATCCTTGTAATCGATCCTGCTACCTGTGATGCCGAACAACT
APOE_qPCR Foward	GGGTCGCTTTTGGGATTACCTG
APOE_qPCR Reverse	CAACTCCTTCATGGTCTCGTCC
APOC1_qPCR Foward	AGGACAAGGCTCGGGAACTCAT
APOC1_qPCR Reverse	GATGTCACCCTTCAGGTCCTCA
GAPDH_qPCR Foward	GTCTCCTCTGACTTCAACAGCG
GAPDH_qPCR Reverse	ACCACCCTGTTGCTGTAGCCAA
NR5A2_qPCR Forward	GGCTTATGTGCAAAATGGCAGATC
NR5A2_qPCR Reverse	GCTCACTCCAGCAGTTCTGAAG
HOXA13_qPCR Foward	TGGAACGGCCAAATGTACTGCC
HOXA13_qPCR Reverse	GGTATAAGGCACGCGCTTCTTTC
NR1I2_qPCR Forward	GCTGTCCTACTGCTTGGAAGAC
NR1I2_qPCR Reverse	CTGCATCAGCACATACTCCTCC
HNF4A_qPCR Forward	GGTGTCCATACGCATCCTTGAC
HNF4A_qPCR Reverse	AGCCGCTTGATCTTCCCTGGAT
CDX1_qPCR Forward	GAGAAGGAGTTTCATTACAGCCG
CDX1_qPCR Reverse	GTTCACTTTGCGCTCCTTTGCC

Supplementary Figure

Supplementary Figure 1

a

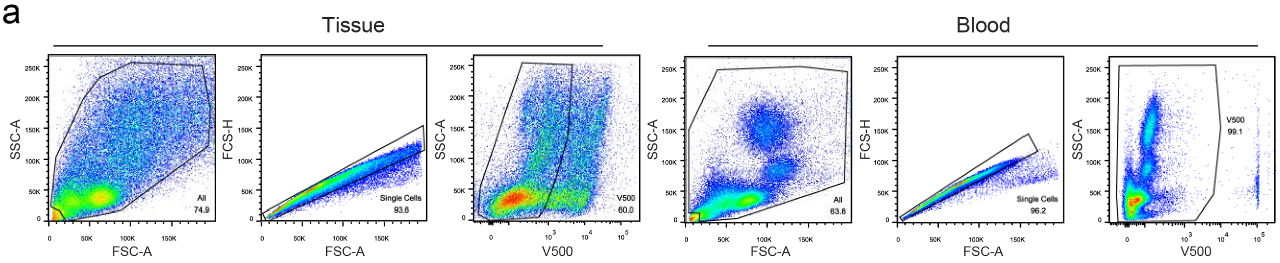

b

Patient ID	Number of cell				*Hp*	Lauren's classification
	Tumor	Paratumor	Blood	TIL		
GC01	4040	NA	NA	NA	Unkown	Mixed
GC02	3628	NA	NA	8924	N	Mixed
GC03	4332/ 3733	5538	NA	NA	N	Diffuse
GC04	5586	4561	NA	NA	P	Mixed
GC05	4229	3694	NA	NA	N	Mixed
GC06	6303	9303	1457	NA	N	Diffuse
GC07	6239	1988	8159	NA	N	Intestinal
GC08	7457/ 2448	4625	7252	NA	N	Intestinal
GC09	6303	6183	NA	NA	P	Mixed
GC10	8820	13893/ 12167	7267	8404	P	Mixed

c

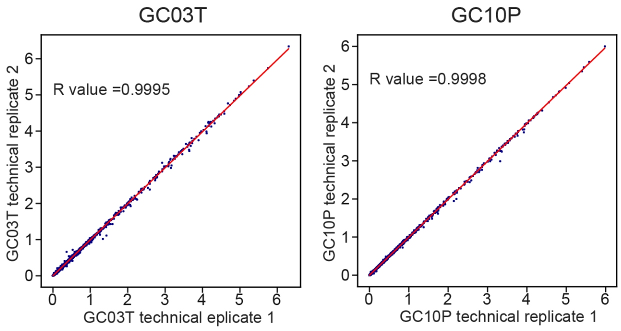

d e f

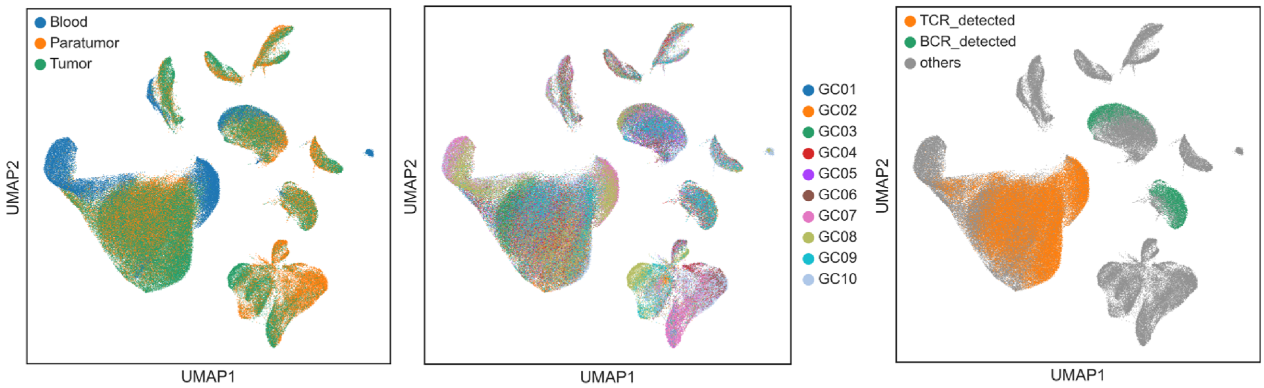

Supplementary Figure 1. Profiling gastric cancer tumor microenvironment by scRNA-seq.
(a) FACS gating strategy of live cells sorting for solid tissue (left panel) and blood (right panel).

(b) Number of cells of each sample type and clinical information collected from each patient. See Supplementary Data 1 for more details. *Hp*, *Helicobacter pylori*; N and P represent negative and positive, respectively.

(c) Scatter plot of average gene expression in replicates. Each point represents one gene. Pearson correlations between replicates were calculated.

(d-f) UMAP of all 166,533 single cells colored by cell origin (e), patient identity (f), and TCR/BCR detection (g).

Supplementary Figure 2

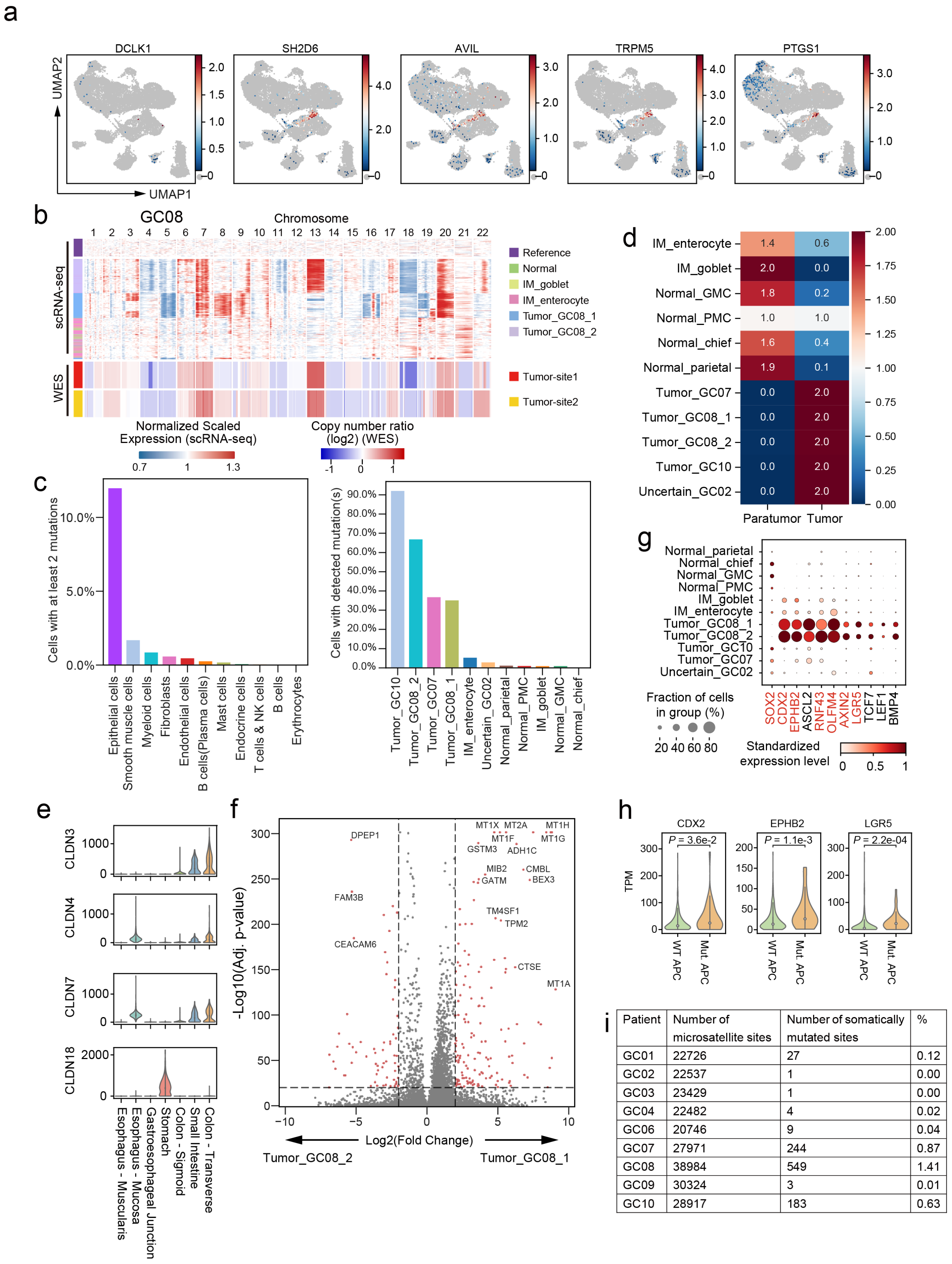

Patient	Number of microsatellite sites	Number of somatically mutated sites	%
GC01	22726	27	0.12
GC02	22537	1	0.00
GC03	23429	1	0.00
GC04	22482	4	0.02
GC06	20746	9	0.04
GC07	27971	244	0.87
GC08	38984	549	1.41
GC09	30324	3	0.01
GC10	28917	183	0.63

Supplementary Figure 2. Characteristics of epithelial cell subsets and malignant state.

(a) UMAPs showing the expression of reported marker genes of tuft cells in epithelial cells.

(b) Inferred CNV profiles of epithelial cells of a representative patient (GC08) based on the scRNA-seq dataset and the whole-exome sequencing (WES) dataset. Red, amplifications; blue, deletions.

(c) Bar plot showing mutation distribution in main cell clusters (left) and epithelial cell clusters (right).

(d) Tissue preference of each epithelial cluster in Fig. 2d estimated by Ro/e score.

(e) Violin plot showing the expression of CLDN-related genes across gastrointestinal tract samples from the GTEx dataset.

(f) Volcano plot showing differentially expressed genes between Tumor_GC08_1 and Tumor_GC08_2. Dotted lines indicate p-value < 1e-20 and |log2 (FC)| > 2. (two-sided Wilcoxon rank-sum test)

(g) Dot plot showing the expression of Wnt-related genes. Dot size indicates the proportion of expressing cells, colored by standardized expression levels. Genes in red indicated a low cellular detection rate outside epithelial cells.

(h) Violin plot showing the expression of *CDX2*, *EPHB2* and *LGR5* in patients with or without APC mutation from TCGA-STAD dataset (two-sided Wilcoxon rank-sum test).

(i) Table showing the MSI status predicted by MSIsensor using the WES data.

Supplementary Figure 3

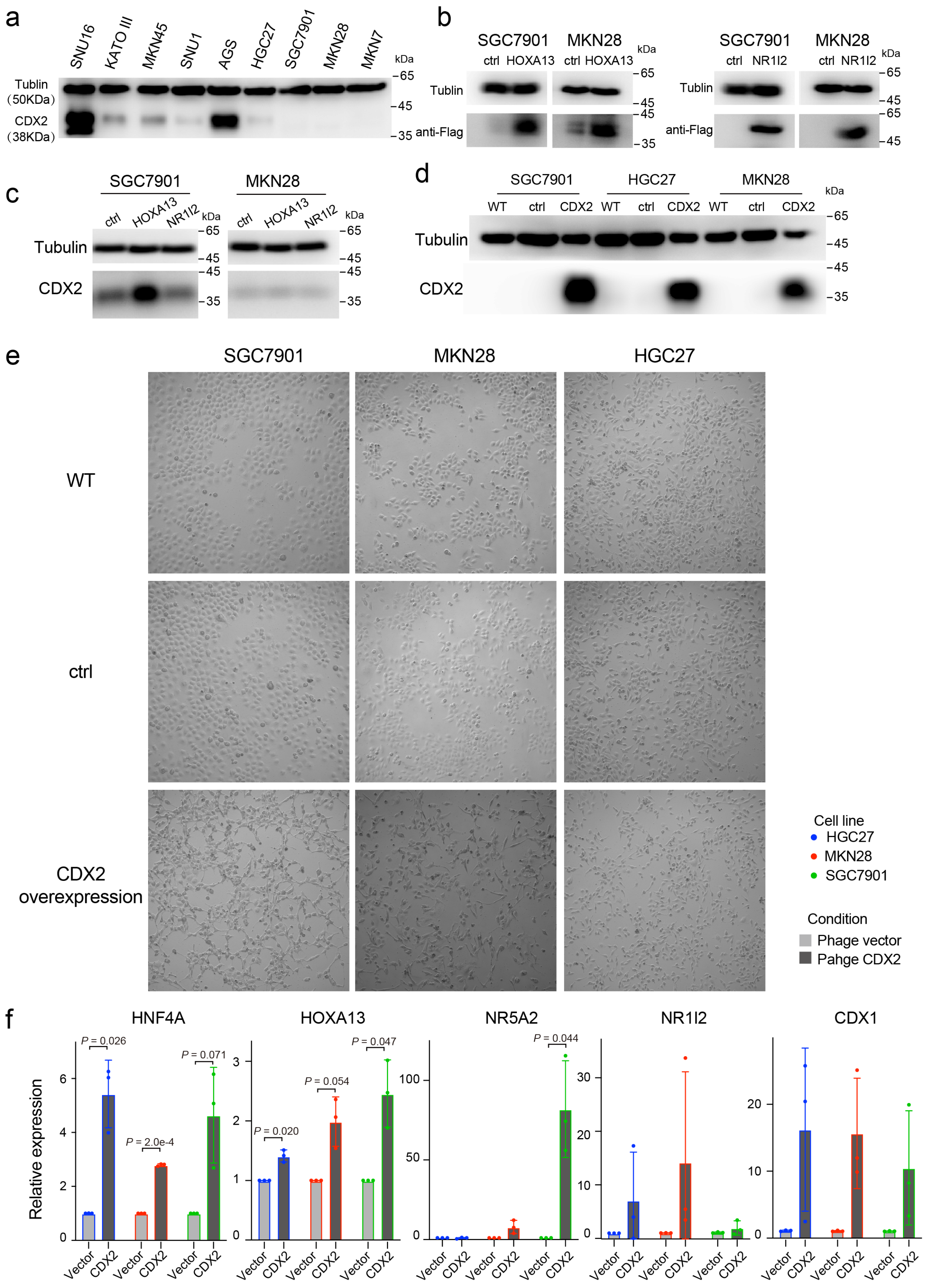

Supplementary Figure 3. The effect of CDX2 overexpression in gastric cancer cell lines.

(a) Baseline expression of nine gastric cancer cell lines, including SNU-16, KATO III, MKN-45, SNU-1, AGS, SGC-7901, HGC-27, MKN-28 and MKN-7,were measured by western blot. The experiments were repeated three times, and here a representative result was shown.

(b) Western blot analysis to confirm the overexpression of HOXA13 and NR1I2 in SGC-7901 and MKN-28 infected with negative control (ctrl), HOXA13, or NR1I2 overexpression lentivirus. The experiments were repeated three times, and here a representative result was shown.

(c) Western blot analysis to detect the expression of CDX2 in SGC-7901 and MKN-28 infected with negative control (ctrl), HOXA13, or NR1I2 overexpression lentivirus. The experiments were repeated three times, and here a representative result was shown.

(d) Western blot analysis to confirm the overexpression of CDX2 in SGC-7901, MKN-28 and HGC-27 infected with negative control (ctrl) or CDX2 overexpression lentivirus. WT, wildtype cell line. The experiments were repeated three times, and here a representative result was shown.

(e) Effect of CDX2 overexpression on SGC-7901, HGC-27, MKN-28 morphology. The experiments were repeated three times, and here a representative result was shown.

(f) The expressions of HNF4A, HOXA13, NR5A2, NR1I2 and CDX1 were measured in SGC-7901, MKN-28 and HGC-27 infected with negative control (ctrl) and CDX2 overexpression lentivirus by qPCR. Each column represents the mean ± SD of three independent experiments (n = 3) (two-sided Student’s t test).

Supplementary Figure 4

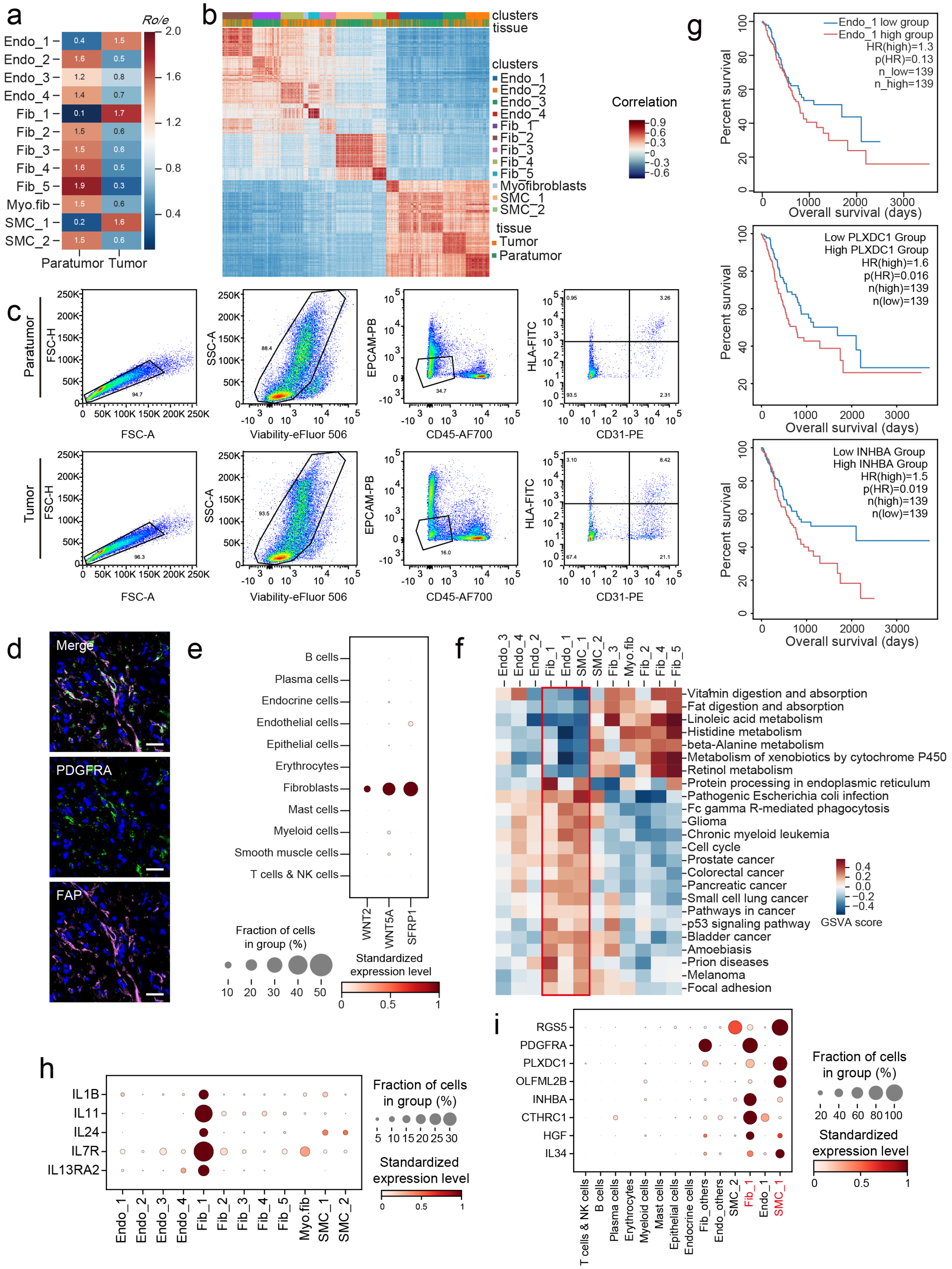

Supplementary Figure 4. Distinct stromal cell subsets and their expression signatures.

(a) Tissue preference of each stromal cell subset in Fig. 3b estimated by Ro/e score.

(b) Pairwise correlation of all stromal cells. Each pixel in the heatmap represents the level of correlation between two cells (the corresponding row and column).

(c) FACS gating strategy of MHC class II$^+$ endothelial cells from tumor and paratumor tissues of GC patients.

(d) Multicolor IHC staining with anti-PDGFRA and anti-FAP antibodies showing FAP$^+$ fibroblasts (n=6). The scale bar represents 20 μm.

(e) Dot plots showing WNT-related genes for clusters in Fig. 1b. Dot size indicates the proportion of expressing cells, colored by standardized expression levels.

(f) Differences in KEGG pathways and GO terms activities scored by GSVA among the different stromal cell subtypes.

(g) High-level *PLXDC1* and *INHBA* were associated with poor overall survival in the TCGA-STAD cohort. Kaplan–Meier curves of overall survival when stratifying the patients by high (top 40%) and low (bottom 40%) expression of the respective gene set. HR (hazard ratio) and p(HR) was calculated by a Cox's proportional hazard model.

(h) Dot plots showing the expression of interleukin-related genes in stromal cell clusters. Dot size indicates the proportion of expressing cells, colored by standardized expression levels.

(i) Dot plots showing the expression of survival-related genes in main cell clusters. Dot size indicates the proportion of expressing cells, colored by standardized expression levels.

Supplementary Figure 5

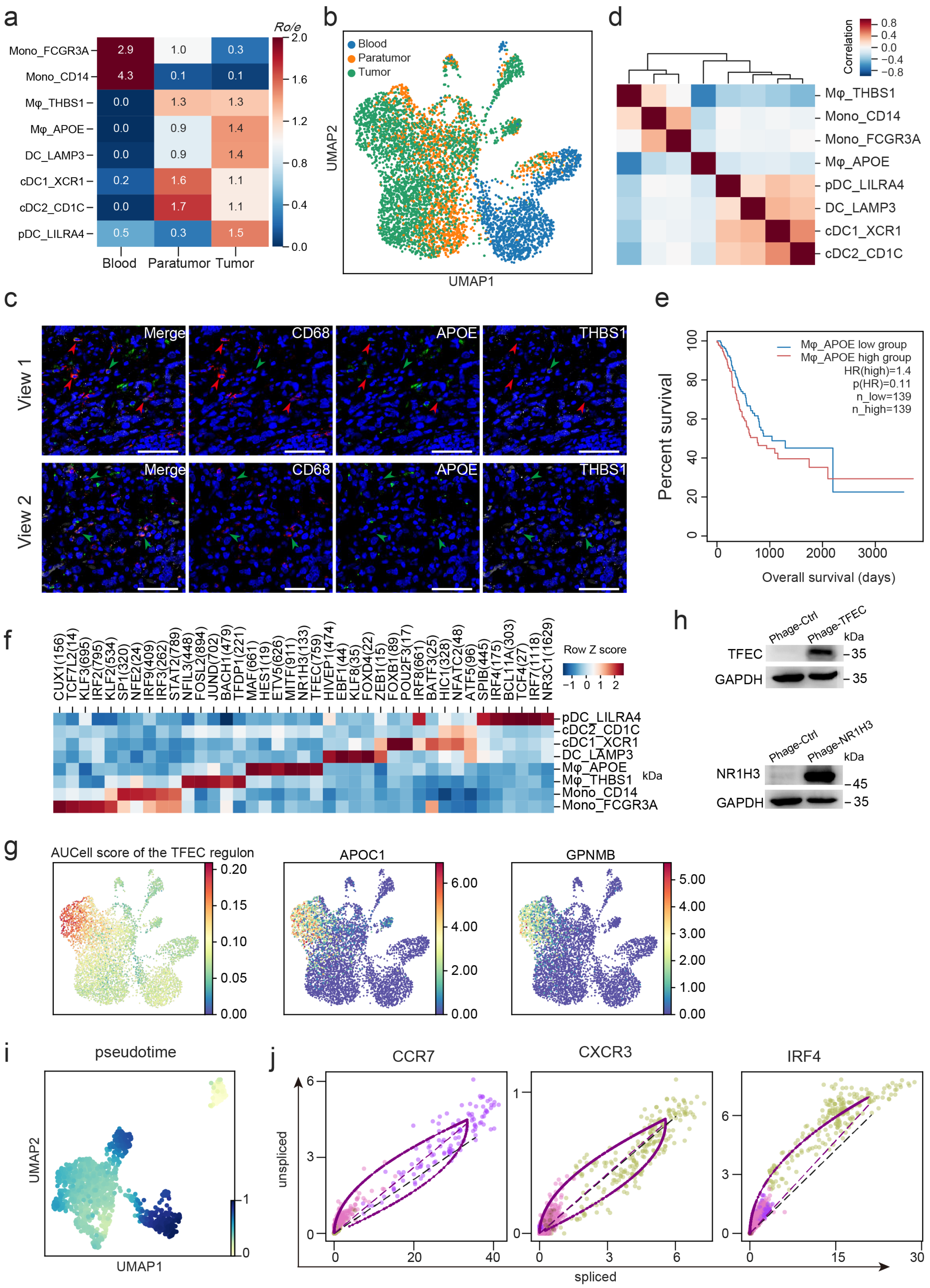

Supplementary Figure 5. Characterization of myeloid cells in GC.

(a) Tissue preference of each myeloid cell subset from Fig. 4a estimated by Ro/e score.

(b) UMAP of stromal cells colored by cellular tissue origin.

(c) Multicolor IHC staining with anti-CD68, anti-APOE and anti-THBS1 antibodies (n=6). The red and green arrows indicate $CD68^{+}APOE^{+}$ and $CD68^{+}THBS1^{+}$ macrophage, respectively. The scale bar represents 50 μm.

(d) Heatmap of Pearson correlation between myeloid cell subsets based on 1000 highly variable genes.

(e) Kaplan–Meier curves of overall survival when stratifying the patients by high (top 40%) and low (bottom 40%) proportion of Mφ_APOE in TCGA-STAD dataset. HR (hazard ratio) and p(HR) was calculated by a Cox's proportional hazard model.

(f) Heatmap of AUCell scores of TF regulon activity calculated by SCENIC for myeloid cell subsets.

(g) UMAP of myeloid cells, colored by the AUCell scores of the TF regulon activity of TFEC, or by the normalized expression of genes.

(h) THP-1 cells were transduced with negative control (ctrl), NR1H3 overexpression, or TFEC overexpression virus, followed by western blot analysis to determine the protein expression of NR1H3 or TFEC. GAPDH is the loading control. The experiments were repeated three times, and here a representative result was shown.

(i) Diffusion map showing the pseudotime of DCs, which was calculated according to RNA velocity.

(j) Velocity analysis of the spliced and unspliced mRNAs of *CCR7*, *CXCR3*, and *IRF4* in DCs. Each dot represents one cell.

Supplementary Figure 6

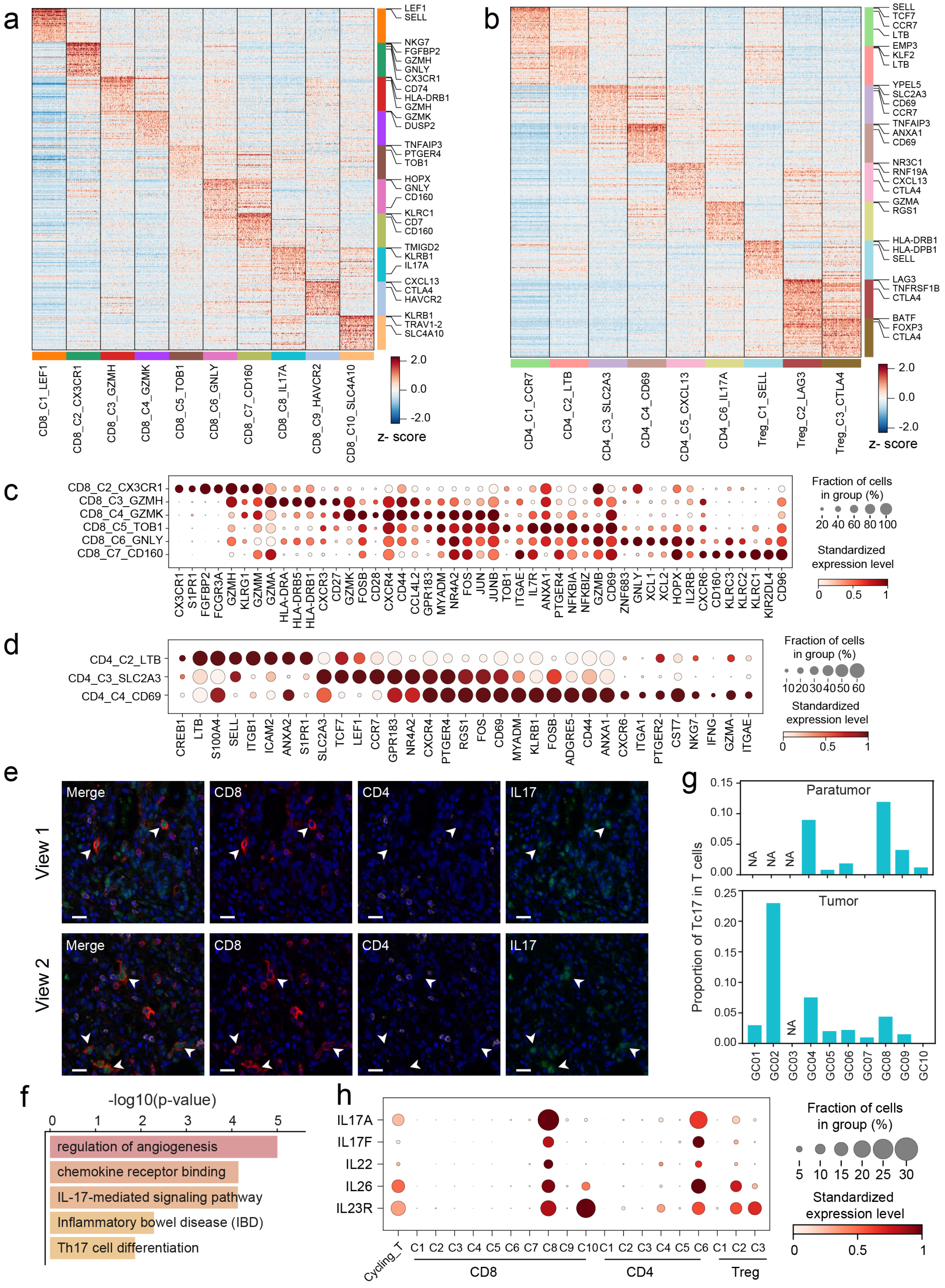

Supplementary Figure 6. Characterization of T-cell subsets based on expression profiling and tissue origin.

(a) Heatmap showing the expression of differentially expressed genes (rows) across inferred $CD4^+$ T cell types (columns), with canonical marker genes highlighted.

(b) Heatmap showing the expression of differentially expressed genes (rows) across inferred $CD8^+$ T cell types (columns), with canonical marker genes highlighted.

(c, d) Dot plots showing marker genes across different T cell subsets. Dot size indicates the proportion of expressing cells, colored by standardized expression levels.

(e) Multicolor IHC staining with anti-CD4, anti-CD8, and anti-IL17A antibodies, exemplified by patient GC988419 (n=6). The white arrows indicate $CD8^+IL17^+$ T cells. The scale bar represents 20 μm.

(f) Bar plots of the KEGG terms or pathways enriched for highly expressed genes in Tc17 (top) and Th17 (bottom). *P*-values were calculated by the hypergeometric test.

(g) Bar plots showing the proportion of Tc17 cells in the paratumor (left) and the tumor (right) of 10 GC patients. NAs represent unavailable data.

(h) Dot plots showing interleukin-related genes specifically expressed by Th17 and Tc17. Dot size indicates the proportion of expressing cells, colored by standardized expression levels.

Supplementary Figure 7

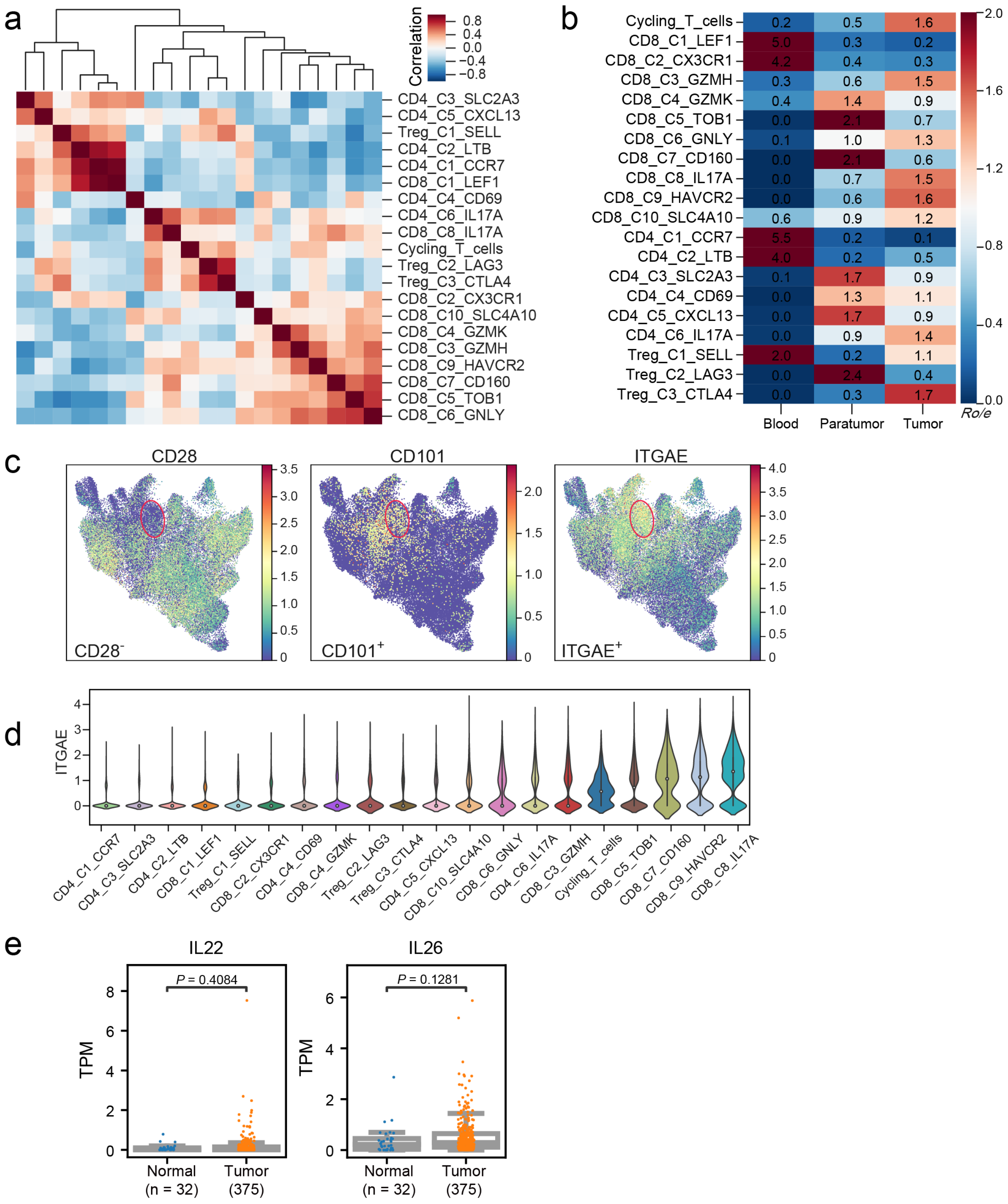

Supplementary Figure 7. Characterization of T-cell subsets based on expression profiling and tissue origin.

(a) Heatmap of Pearson correlation between T cell subsets based on 1000 highly variable genes.

(b) Tissue preference of each T cell cluster in Fig. 5a estimated by Ro/e score.

(c) UMAP showing the expression of *CD28*, *CD101*, and *ITGAE* in T cells.

(d) Violin plot showing the expression of *ITGAE* in T-cell subsets.

(e) Boxplot showing the expression of *IL22* and *IL26* in tumor (n=375) and normal tissue (n=32) from TCGA-STAD data (two-sided Wilcoxon rank-sum test). For all boxplots in this paper: box, interquartile range (IQR); horizontal line, median; whiskers, most extreme values within ±1.5 × IQR.

Supplementary Figure 8

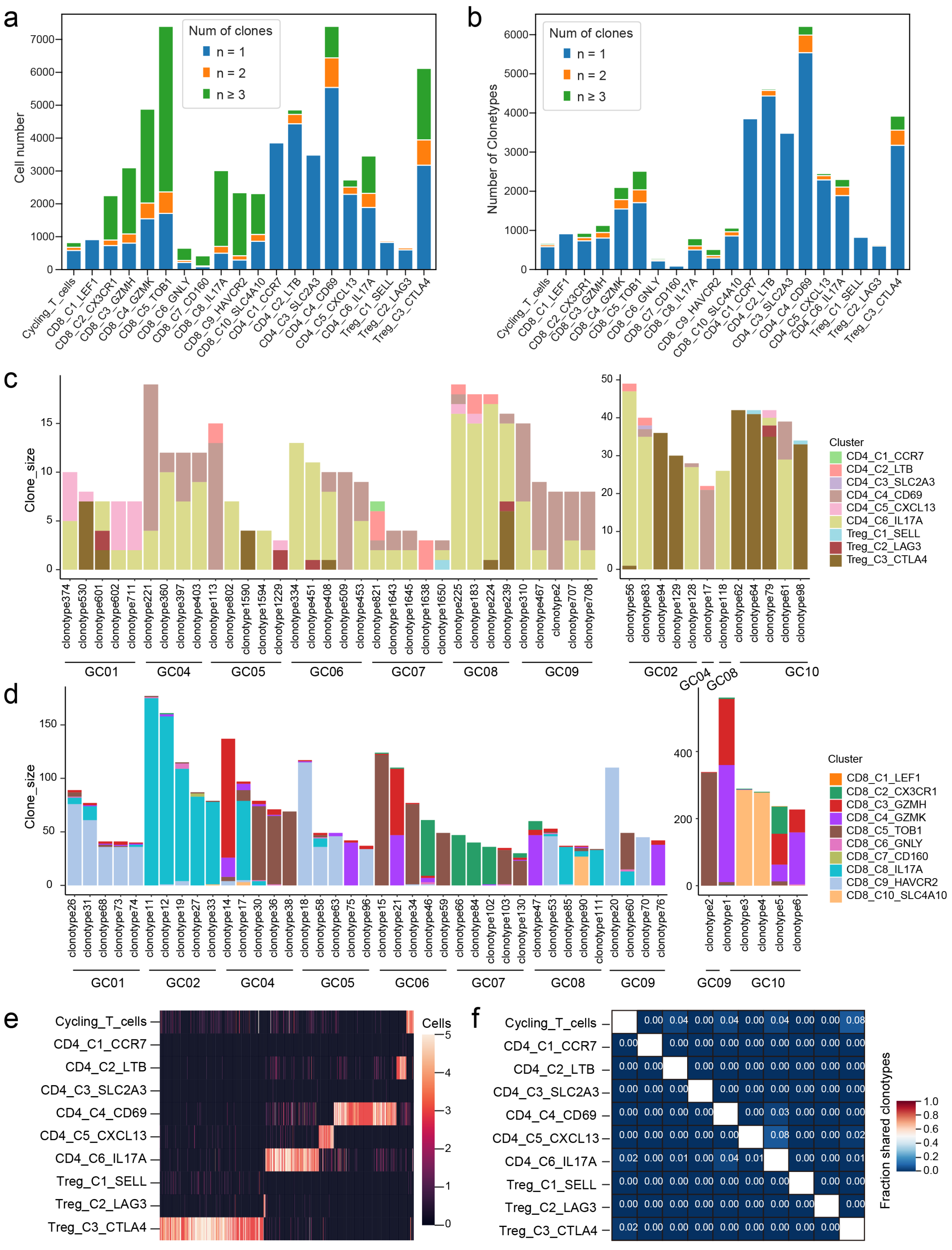

Supplementary Figure 8. TCR clone information of T cell subsets and TCR sharing in CD4$^+$ T Cells.

(a-b) Bar plots showing the number of clonal cells (a) and clonotypes (b) in each T cell subset. The clonotypes are categorized as unique (n = 1) or clonal (n ≥ 2) based on their cell numbers.

(c-d) CD8$^+$ (c) and CD4$^+$ (d) cluster composition of the top five most abundant clones for each patient. Each bar is colored by proportion of cell clusters within the clone. Cells belonging to a unique clone preferentially accumulate in one cluster.

(e) The distribution of clonotypes in CD4$^+$ T cell subsets and cycling T cells. Lighter color indicates higher cell number; cell numbers were capped at 5.

(f) Heatmap showing the fraction of clonotypes belonging to a primary phenotype cluster (rows) that are shared with other secondary phenotype clusters (columns).

Supplementary Figure 9

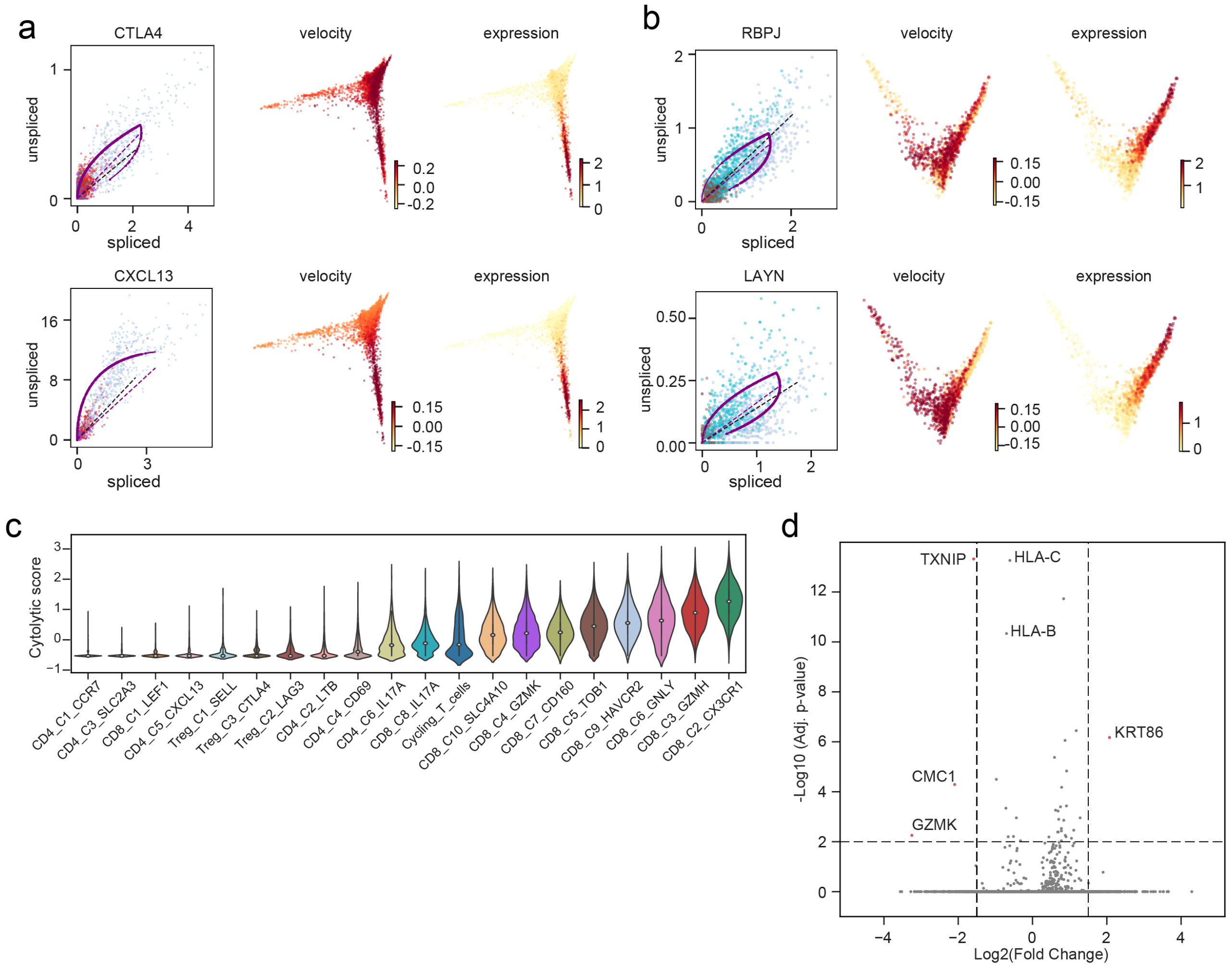

Supplementary Figure 9. Example genes in velocity analysis of the two exhaustion trajectories and gene expression signatures of Tc17 cells.

(a) Velocity analysis of the spliced and unspliced mRNAs of *CTLA4* and *CXCL13* in cytolytic-exhaustion trajectory. Each dot represents one cell.

(b) Velocity analysis of the spliced and unspliced mRNAs of *RBPJ* and *LAYN* in Tc17-exhaustion trajectory. Each dot represents one cell.

(c) Violin plot showing the cytolytic score in T-cell subsets.

(d) Volcano plot showing differentially expressed genes between Tc17-trajectory-derived exhausted $CD8^+$ T cells and cytolytic-trajectory-derived exhausted $CD8^+$ T cells. Dotted lines indicate adjusted *p*-value < 0.01 and |log2 (FC)| > 1.5. (two-sided Wilcoxon rank-sum test with Bonferroni correction)

Supplementary Figure 10

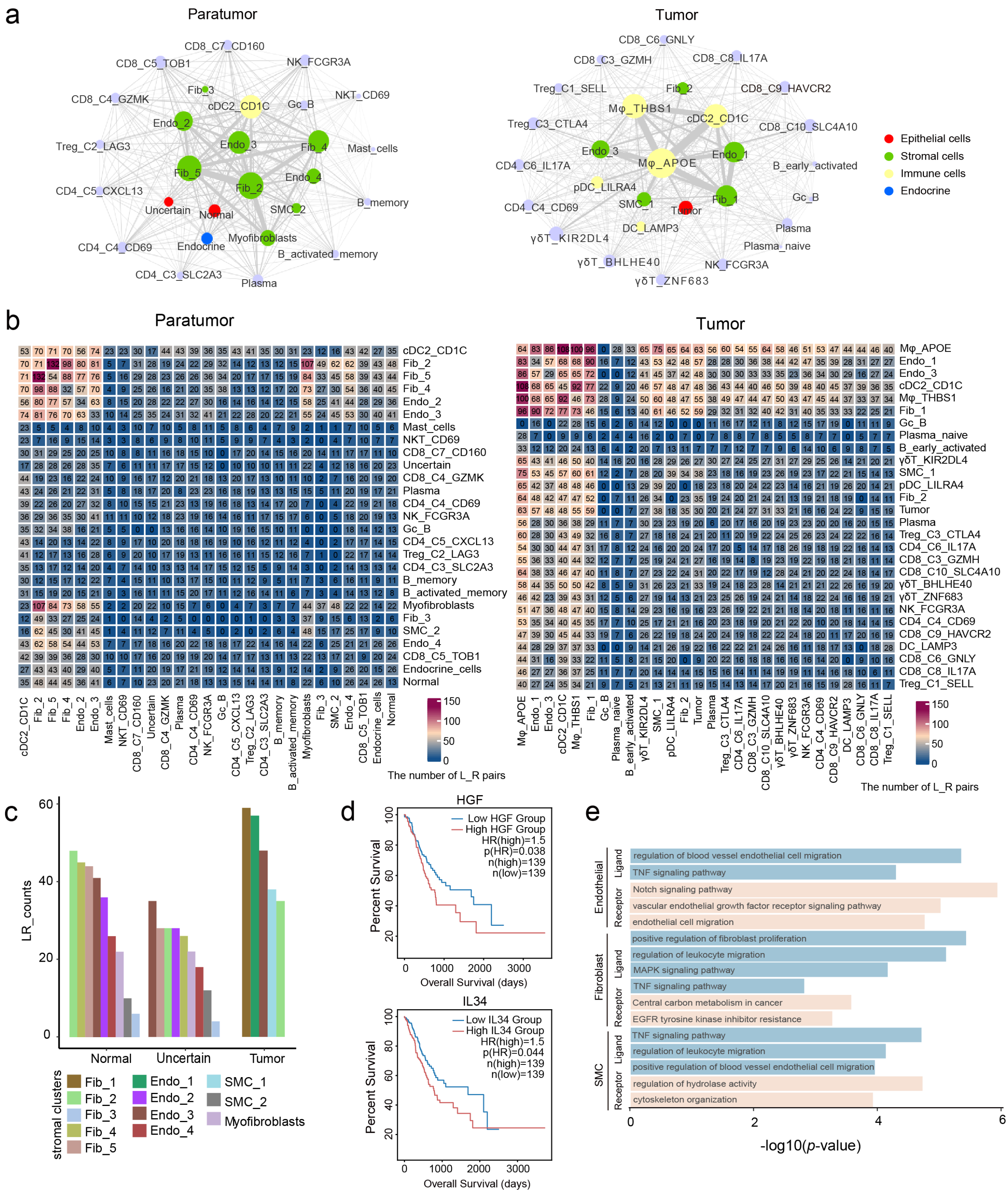

Supplementary Figure 10. Predicted cell-cell interactions based on ligand-receptor interactions.

(a) Interaction networks among cellular subsets in paratumor (left) and tumor (right) samples. Nodes represent cell clusters in a network, and the size of the node indicates the total number of interactions that were significant (p-value < 0.01) in at least one patient with other cell subsets. Edges connect pairs of cell subsets, and the edge thickness indicates the relative number of interactions between the connected subsets.

(b) Heatmap depicting the number of all possible interactions between the clusters analyzed. The interactions that were significant (p-value < 0.01) in at least one patient were counted.

(c) The number of L-R interactions between epithelial cell subsets and stromal cell subsets.

(d) High-level *HGF* and *IL34* were associated with poor overall survival in the TCGA-STAD cohort. Kaplan–Meier curves of overall survival when stratifying the patients by high (top 40%) and low (bottom 40%) expression of the respective gene set. HR (hazard ratio) and p(HR) was calculated by a Cox's proportional hazard model.

(e) GO terms and KEGG pathway enrichment analysis using the ligand or receptor genes of Endo_1, Fib_1 and SMC_1 with other subsets in tumors. *P*-values were calculated by the hypergeometric test.

Supplementary Figure 11

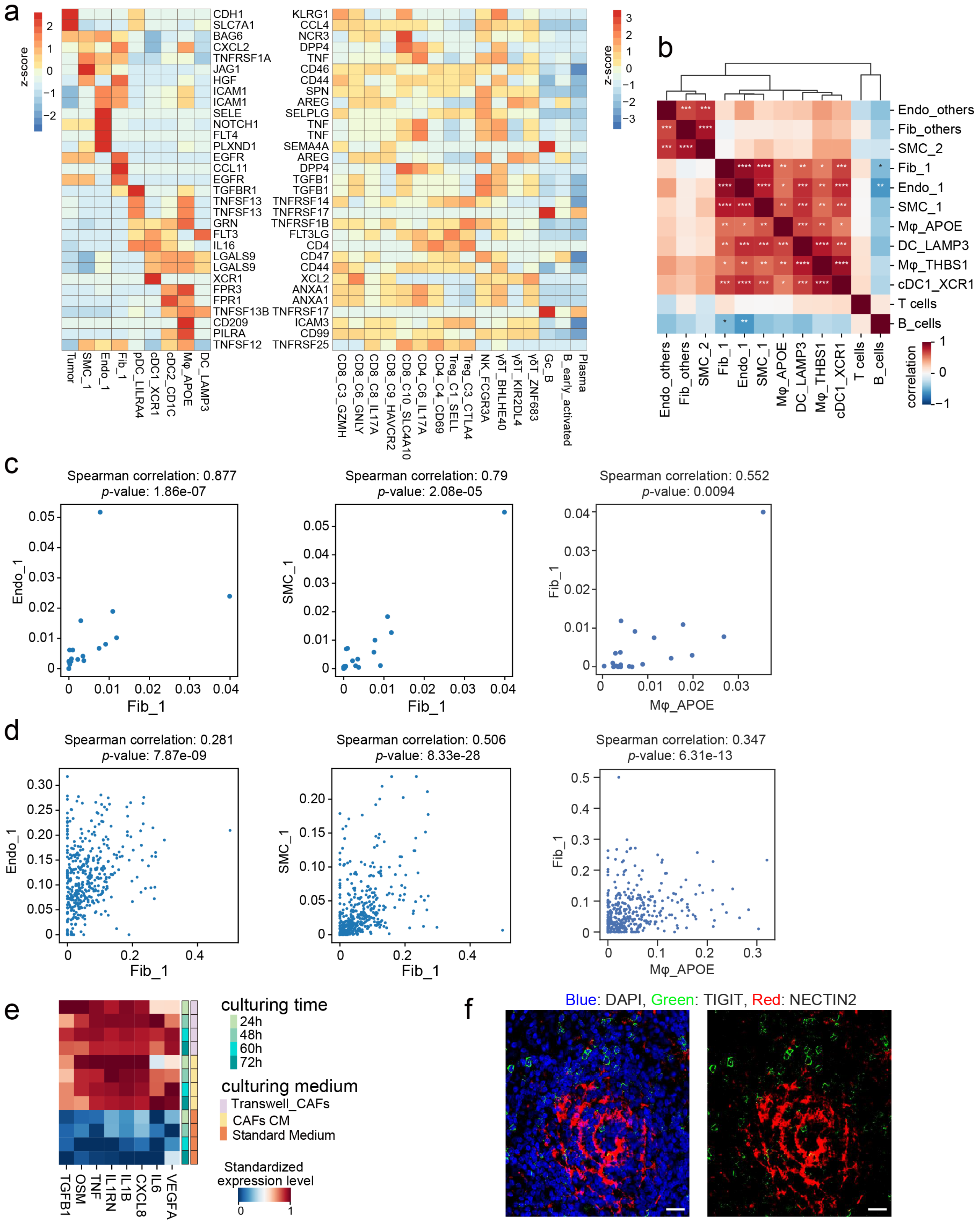

Supplementary Figure 11. Predicted cell-cell interactions based on ligand-receptor interactions.

(a) Heatmap of scaled expression of selected ligand-receptor pairs between lymphocyte subsets (right) and other subsets (left) in tumors.

(b) Heatmap showing the Spearman's rank correlation coefficients between the inferred proportions of different cell types in single-cell dataset. *$P < 0.05$, ** $P < 0.01$, *** $P < 0.001$, and **** $P < 0.0001$. *P*-value were calculated by two-sided t-test and the exact values can be found in the Source Data.

(c-d) Scatterplot showing the Spearman's rank correlation coefficients of cell fractions between Fib_1 and Endo_1/SMC_1/ Mφ_APOE in the scRNA-seq dataset (c) and the TCGA-STAD dataset (d) (two-sided t-test).

(e) Heatmap showing the mean expression of cytokines in THP-1 monocyte-derived macrophages that were co-cultured with gastric CAFs in a transwell system, cultured by gastric CAFs conditioned mediums (CM), or cultured by standard medium for 24 h, 48 h, 60 h, 72 h.

(f) Multicolor IHC staining with anti-TIGIT and anti-NECTIN2 antibodies, exemplified by patient GC771257 (n=6). The scale bar represents 20 μm.

Supplementary Figure 12

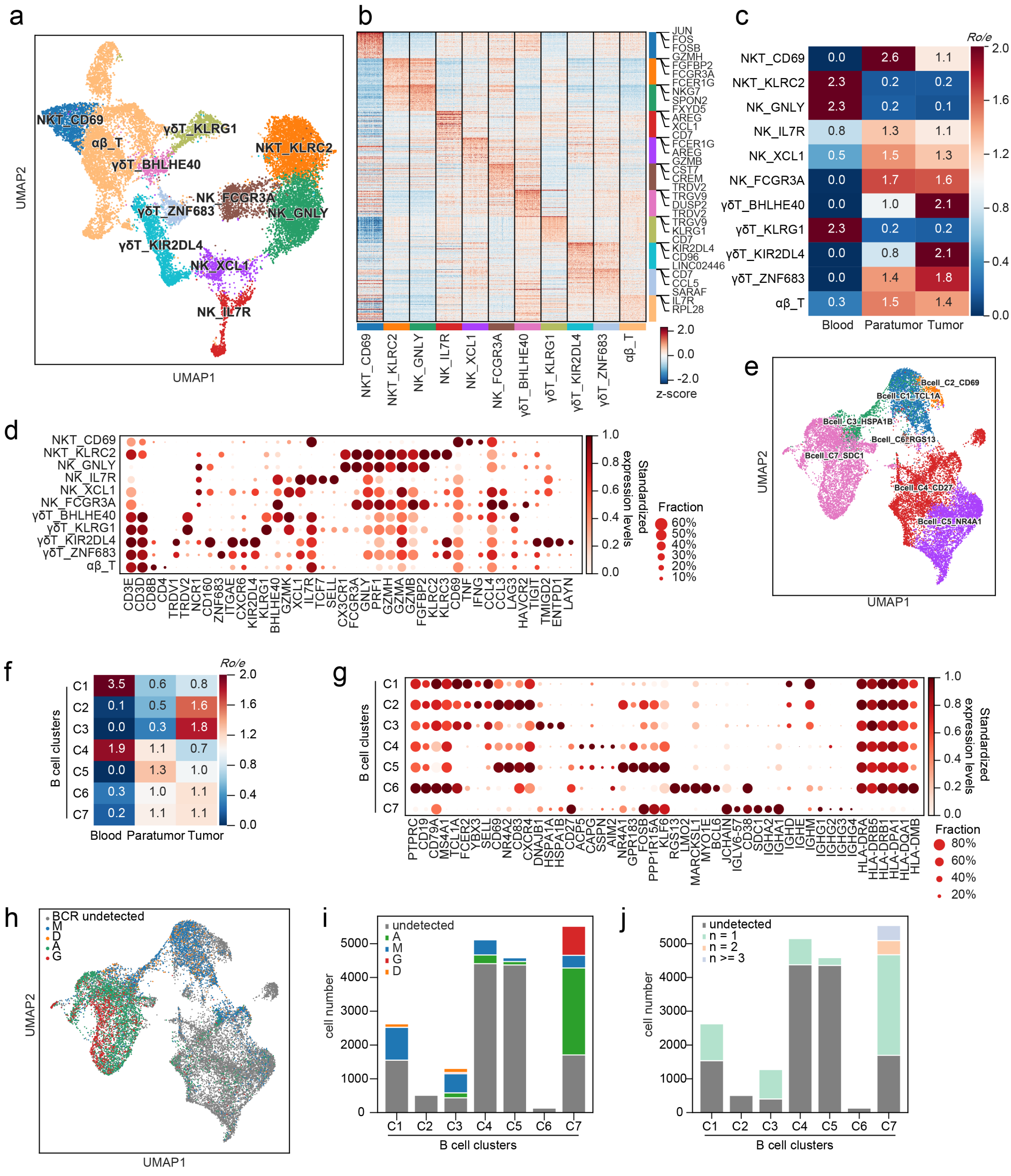

Supplementary Figure 12. Cluster characterization of NK and B cells in GC.

(a) UMAP of the remaining NK&T cells lacking TCR information. Clusters are labeled with inferred cell types.

(b) Heatmap showing the differentially expressed genes (rows) across inferred cell types (columns).

(c) Tissue preference of each subset from (a) estimated by Ro/e score.

(d) Dot plots showing marker genes for T cells and NK cells. Dot size indicates the proportion of expressing cells, colored by standardized expression levels.

(e) UMAP of B cells. Clusters are labeled with inferred cell types.

(f) Tissue preference of each B cell subset from (e) estimated by Ro/e score.

(g) Dot plots showing marker genes across B cell subsets. Dot size indicates the proportion of expressing cells, colored by standardized expression levels.

(h) UMAP of B cells colored by different antibody isotypes.

(i) Bar plot showing the number of different antibody isotypes in each B cell subset.

(j) Bar plots showing the number of clonal cells in each B cell subset.

Supplementary Figure 13

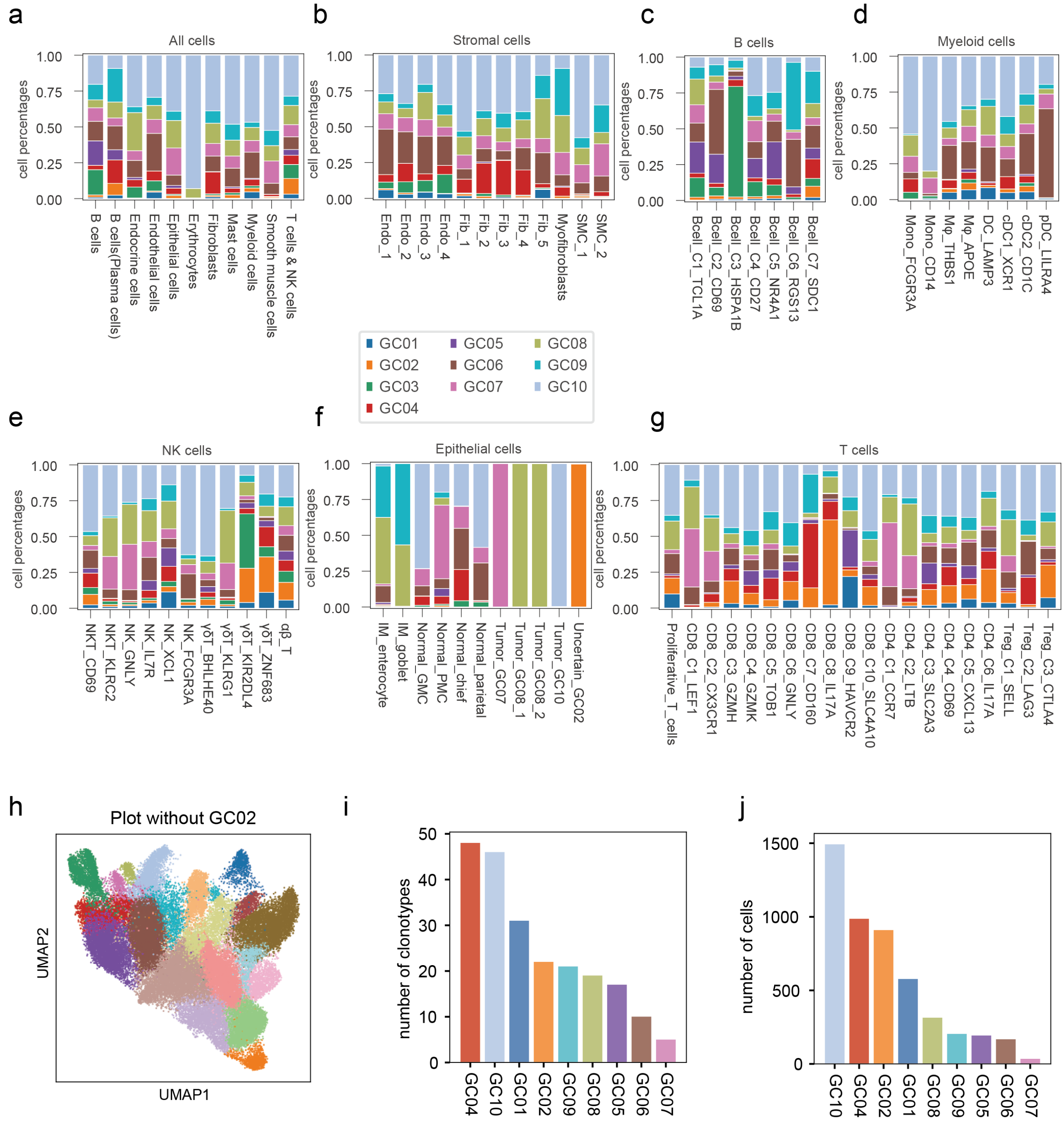

Supplementary Figure 13. The evaluation of patient effects.

(a-g) Stacked bar plot showing the patient distribution of each cluster.

(h) UMAP plot showing the T cells without GC02.

(i-j) Bar plots showing the number of clonotypes (i) and the number of cells (j) contributed by each patient in the cell subset of Tc17 trajectory analysis.

Supplementary Figure 14

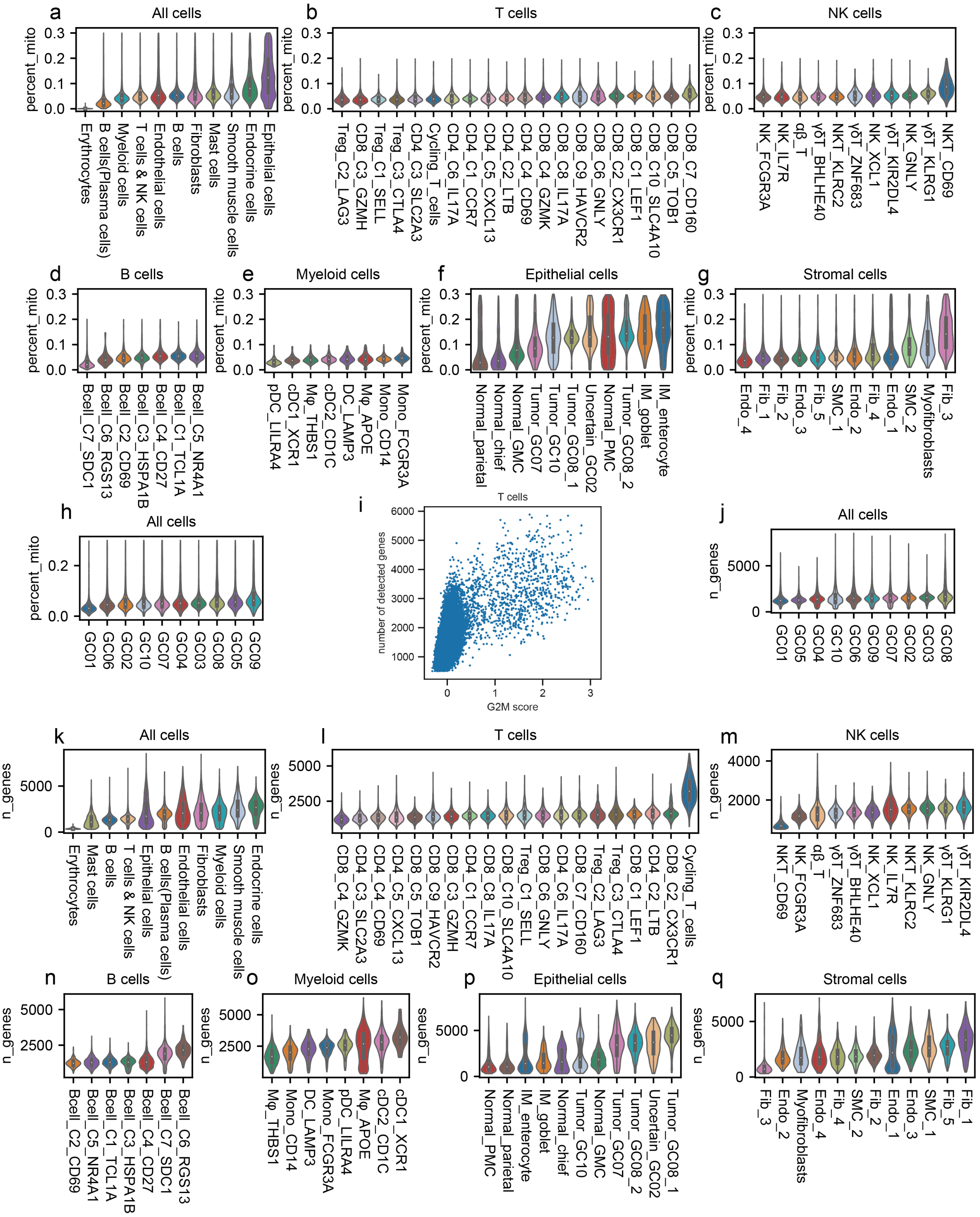

Supplementary Figure 14. The evaluation of cell quality difference.

(a-g) Violin plot showing the distribution of mitochondrial gene percentages in each cluster

(h) Violin plot showing the distribution of mitochondrial gene percentages in each patient.

(i) Scatter plot showing a positive correlation between the G2M score and the number of detected genes. Each dot represents a single cell.

(j) Violin plot showing the distribution of the number of detected genes in each patient.

(k-q) Violin plot showing the distribution of the number of detected genes in each cluster.

Supplementary Figure 15

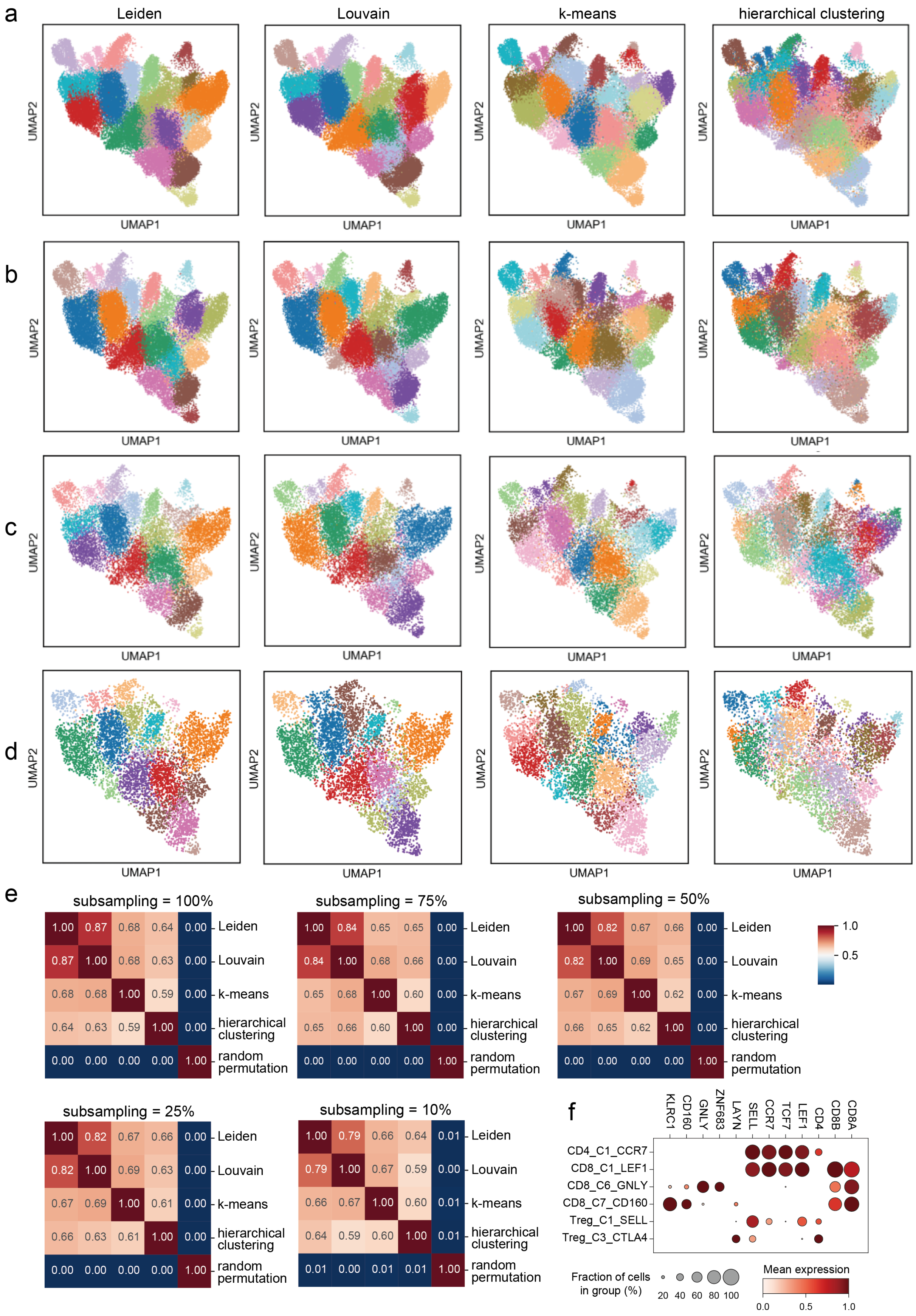

Supplementary Figure 15. The evaluation of the robustness and necessity of T-cell clusters.

(a-d) Down-sampling of T cells to (a) 75%, (b) 50%, (c) 25%, and (d) 10% and clustering with different algorithms.

(e) Heatmaps showing normalized mutual information evaluating the similarity between different clustering results at different subsampling rate.

(f) Dot plot showing selected markers to demonstrate the necessity of distinguishing these clusters: CD4_C1 and CD8_C1; CD8_C6 and CD8_C7; Treg_C1 and Treg_C3.

Supplementary Figure 16

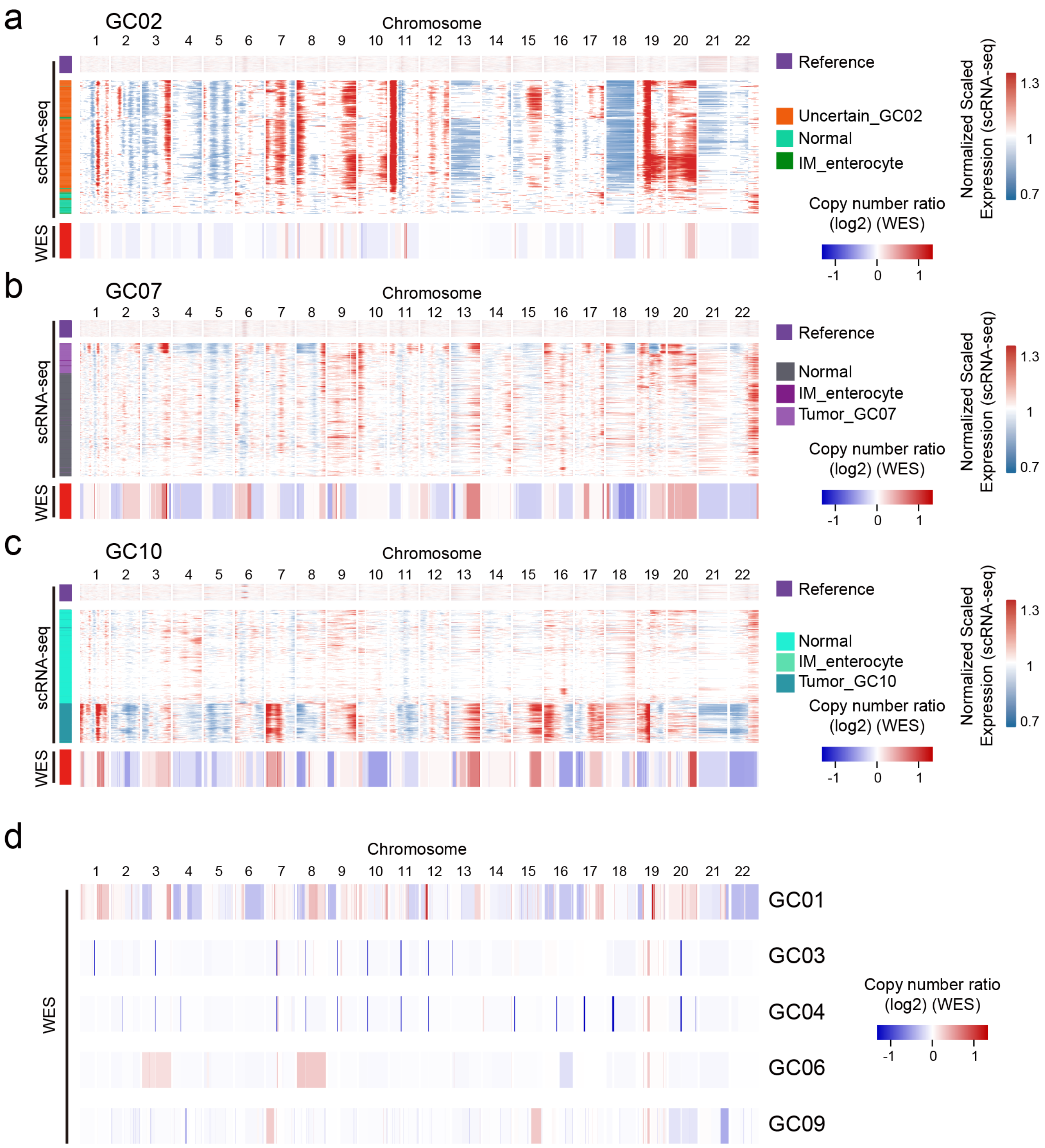

Supplementary Figure 16. Inferred CNV profiles of scRNA-seq dataset and WES.

(a-c) Inferred CNV profiles of epithelial cells based on the scRNA-seq dataset and the WES dataset in GC02 (a), GC07 (b), and GC10 (c). Red, amplifications; blue, deletions.

(d) Inferred CNV profiles based on the WES dataset in GC01, GC03, GC04, GC06 and GC09. Red, amplifications; blue, deletions.

Supplementary Note 1: a systematic evaluation of batch effects

Batch effects can affect clustering and downstream analyses. Firstly, to evaluate the patient-specific features that might affect clustering, we investigated distributions of cell percentages contributed by different patients in each cluster (Supplementary Fig. S13). Most of the clusters had diverse contributors roughly proportional to sample sizes (GC10 had the largest sample size), except for abnormal epithelial cells. Tumor cells were highly heterogeneous thus tumor cells from different patients formed separated clusters; Cells with transcriptional-level intestinal metaplasia (IM) were only detected in several patients, mainly GC08 and GC09, which was also confirmed by bulk RNA-seq (Fig. 2m). Cells in epithelial clusters expressed highly specific marker genes (Fig. 2f, Supplementary Table 1) thus the clustering was not dominated by unwanted batch effects. Besides, clusters consisting largely of cells from blood (e.g., Naïve T cells and Mono_CD14) had fewer contributors as the blood samples only came from GC06, GC07, GC08, GC09, and GC10. As for erythrocytes, very few of them passed through the ACK lysis buffer treatment.

We noticed that GC02 contributed 59% of the cells in CD8_C8_IL17A. We then plot the UMAP without GC02 (Supplementary Fig. 13h) and found the shape of the cluster did not dramatically change. Besides, we counted the number of clonotypes and the number of cells contributed by each patient in the cell subset of Tc17 trajectory analysis (Supplementary Fig. 13i, j) (This subset was used in Figure 6 for the velocity analysis, which consisted of cells that belonged to clonotypes detected in at least two clusters among CD8_C5_TOB1, CD8_C8_IL17A, and CD8_C9_HAVCR2). In this subset, GC02 did not account for a large proportion. These demonstrated that the results were not dominated by GC02 or other patient-specific features.

To evaluate the batch effect caused by cell quality, we first investigated the distributions of percentages of mitochondrial gene counts (abbreviated as “percent_mito” hereafter). The percent_mito raised when the cellular membrane was leaky or disrupted and cytoplasmic RNAs were released, thus it was considered as an indicator for cell quality. The percent_mito of samples from different patients were similar (Supplementary Fig. 14h). We found significantly higher percent_mito in epithelial cells. This phenomenon was found in most of the samples hence it was not accidental, though the reason had not been figured out. A hypothesis was that epithelial cells from stomachs were more vulnerable to the experimental procedures than other cell types. The distributions of percent_mito varied in epithelial subclusters, but this didn’t seriously affect the clustering since each cluster expressed highly specific marker genes (Fig. 2f, Supplementary Table 1). As for plasma cells (named Bcell_C7_SDC1 in B cells clustering), they had lower

percent_mito due to the large number of total UMI counts, which was caused by a remarkable high expression of immunoglobulin-related genes. In the case of NK cells and stromal cells, NKT_CD69 and Fib_3 showed higher percent_mito, which might be affected by the cell-quality effect. Nevertheless, they would not affect the main results as our analyses did not focus on such cells.

The number of detected genes in a cell is another indicator of cell quality. A high percent_mito combined with a low number of detected genes often indicates bad quality. However, biological differences can also influence the number of expressed genes. For example, the number of expressed genes is a robust indicator of developmental potential [2]. Besides, we found cycling cells had higher numbers of genes (Supplementary Fig. 14i, l), which might be caused by the expression of cell-cycle genes like MKI67 (Fig. 5b, c). In addition, tumor cells seemed to express more genes than other cells (Supplementary Fig. 14p).

In conclusion, the clustering was not obviously affected by patient-specific features. Though Fib_3 and NKT_CD69 seemed to be associated with low cell quality as they both had high percent_mito and low numbers of genes, we did not focus on them individually. Other clusters with unusual percent_mito or numbers of genes (e.g., tumor cells and cycling T cells) expressed highly specific marker genes. Therefore, we believed our main results were not significantly affected by batch effects.

Supplementary Note 2: Evaluation of the robustness and necessity of T-cell clusters

We defined 20 clusters in T cells, which might raise doubts about the robustness and necessity of such a complicated clustering. Although some clusters seemed to be very small, the smallest cluster (CD8_C7_CD160) consisted of 420 cells contributed by multiple patient samples (Supplementary Fig. 13g). To evaluate the robustness of the T-cell clusters, we down-sampled the T cells to 75%, 50%, 25%, and 10% of the cell population. PCA was reproduced for these four subsets separately and then UMAP and four clustering approaches (Leiden, Louvain, k-means, and hierarchical clustering) were applied using the first 30 PCs (Supplementary Fig. 15a-d). The results showed that the clusters of the subsampled cells were close to the initial clustering result (Supplementary Fig. 15a-e).

To investigate the necessity of distinguishing some rare clusters, we looked for specifically expressed genes with clear biological significance. CD4_C1 and CD8_C1 were both naïve T cells expressing LEF1, TCF7, CCR7, and SELL (Fig. 5c), while they belonged to $CD4^+$ T cells and $CD8^+$ T cells respectively, which was an important difference. CD8_C6 and CD8_C7 were quite rare clusters, while the former showed distinctive expression of ZNF683 (Tissue-resident T-cell transcription regulator protein) which indicated it was tissue-resident T cells. Besides, GNLY (Granulysin), an antimicrobial peptide, was also highly expressed by CD8_C6 but not by CD8_C7. As for CD8_C7, CD160 and KLRC1 were expressed at a much higher level. In the case of Treg_C1 and Treg_C3, the former expressed much higher levels of LEF1, CCR7, and SELL, indicating a naïve state, while the latter expressed higher levels of exhausted markers like LAYN. The list of cluster-specific genes can also be found in Fig. 5c.

In conclusion, the rare clusters had distinctively expressed genes with evidence of biological significance. Thus, it is necessary to distinguish these clusters instead of mixing them together.

Supplementary Note 3: a case study of GC08 with abnormal Wnt signaling

We found that many genes related to the canonical Wnt signaling, a key oncogenic pathway, were upregulated in tumor cells from Tumor_GC08 compared to the IM clusters (Supplementary Fig. 2g). The expression of *EPHB2*, *LGR5*, *ASCL2* made the tumor cells resemble intestinal stem cells [3, 4]. The expression of *TCF7*, *LEF1*, *BMP4,* and other Wnt target genes suggested an up-regulated Wnt signal. Meanwhile, negative feedback regulators of Wnt signaling, such as *RNF43*, *AXIN2*, were also up-regulated. The WES data showed GC08 had a deletion at chr5:112,840,255, resulting in a frameshift in the last exon of *APC*, which might disrupt the degradation of β-catenin. This variant was not present in the paratumor sample and might be a reason for the up-regulated Wnt signaling in tumor cells. Meanwhile, with the TCGA-STAD dataset, we found *APC* mutation was associated with a higher expression of *CDX2* and stemness-related Wnt targets (Supplementary Fig. S2h). Considering that *CDX2* was reported as a Wnt inhibitor and tumor suppressor in the colon [5, 6], the underlying mechanism of this observation deserves further investigation.

Supplementary References

1. Liu, Y., et al., *Chimeric STAR receptors using TCR machinery mediate robust responses against solid tumors.* Sci Transl Med, 2021. **13**(586).
2. Gulati, G.S., et al., *Single-cell transcriptional diversity is a hallmark of developmental potential.* Science, 2020. **367**(6476): p. 405-411.
3. Merlos-Suárez, A., et al., *The intestinal stem cell signature identifies colorectal cancer stem cells and predicts disease relapse.* Cell Stem Cell, 2011. **8**(5): p. 511-24.
4. Schuijers, J., et al., *Ascl2 acts as an R-spondin/Wnt-responsive switch to control stemness in intestinal crypts.* Cell Stem Cell, 2015. **16**(2): p. 158-70.
5. Yu, J., et al., *CDX2 inhibits the proliferation and tumor formation of colon cancer cells by suppressing Wnt/β-catenin signaling via transactivation of GSK-3β and Axin2 expression.* Cell Death Dis, 2019. **10**(1): p. 26.
6. Bonhomme, C., et al., *The Cdx2 homeobox gene has a tumour suppressor function in the distal colon in addition to a homeotic role during gut development.* Gut, 2003. **52**(10): p. 1465-71.

Supplementary Figure 17. Uncropped Western blots

Supp. Fig 3a. The expression level of CDX2 in gastric cancer cell lines

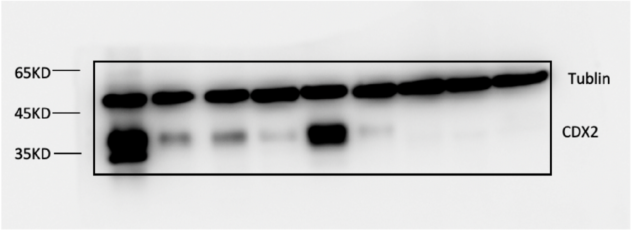

Supp. Fig 3b. The overexpression of HOXA13 and NR1I2 in SGC-7901 and MKN-28

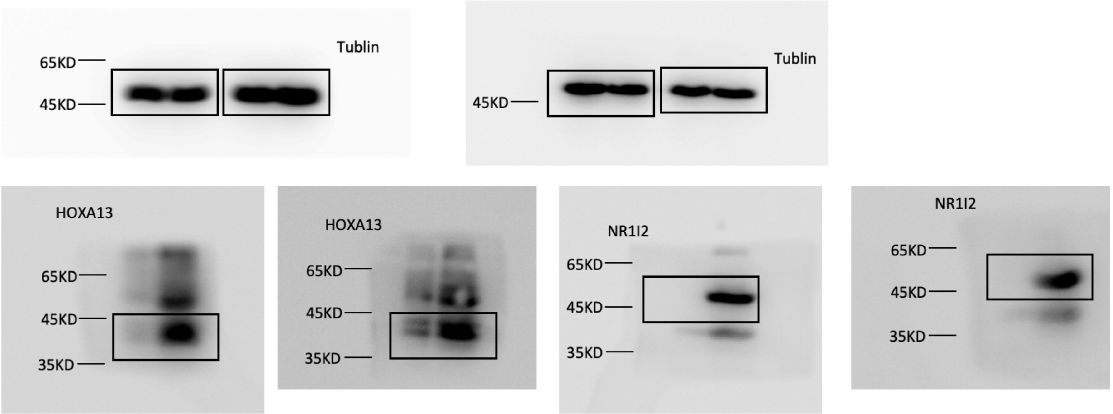

Supp. Fig 3c. The expression of CDX2 in SGC-7901 and MKN-28 infected with negative control (ctrl), HOXA13, or NR1I2 overexpression lentivirus

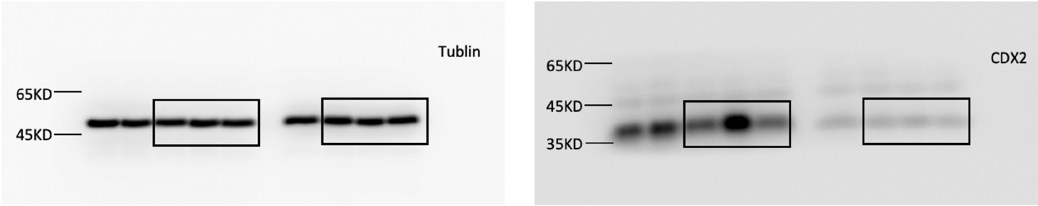

Supp. Fig 3d. The overexpression of CDX2 in SGC-7901, MKN-28 and HGC-27

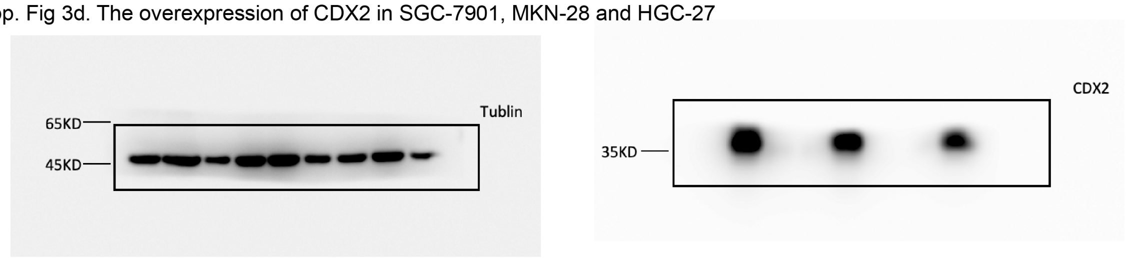

Supplementary Figure 18. Uncropped Western blots

Supp. Fig 5h. The overexpression of NR1H3 and TFEC in THP-1

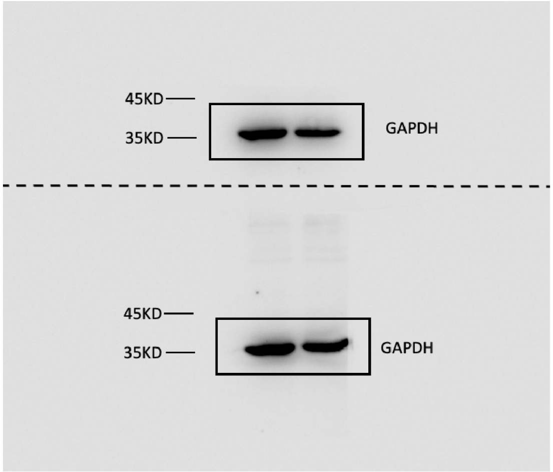

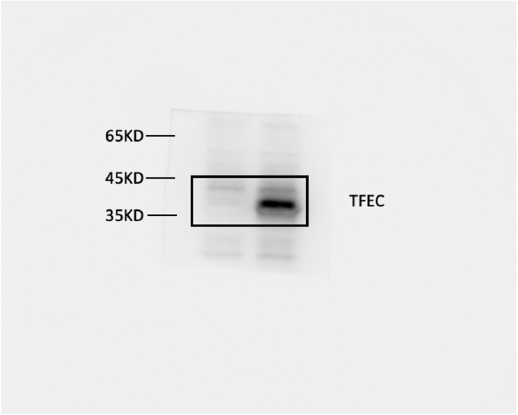

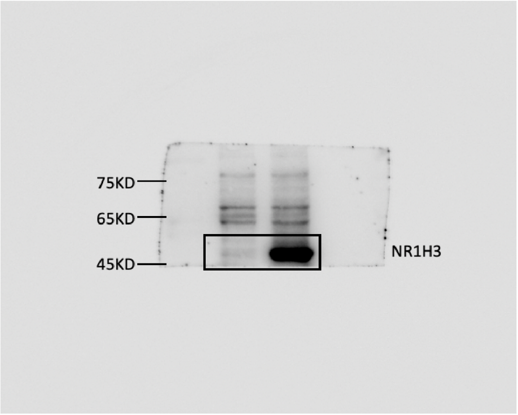

Supplement: Supplementary file 1 — Supplementary Information [file 41467_2022_32627_MOESM1_ESM.pdf]
