# Supplementary material for: scRNA-seq of gastric tumor shows complex intercellular interaction with an alternative T cell exhaustion trajectory
Source: Nat Commun. 2022 Aug 23;13:4943. doi: 10.1038/s41467-022-32627-z (PMC9399107; doi:10.1038/s41467-022-32627-z)
Supplement: Supplementary file 3 — Description of Additional Supplementary Files [file 41467_2022_32627_MOESM3_ESM.pdf]

Description of Additional Supplementary Files

File Name: Supplementary Data 1
Description: Clinical characteristics of GC patients and sequencing informatio

File Name: Supplementary Data 2
Description: The classical markers of each main cell type

File Name: Supplementary Data 3
Description: List of differentially expressed genes in all cell clusters

File Name: Supplementary Data 4
Description: Common genes in both Tc17 and Th17

File Name: Supplementary Data 5
Description: VDJ_gene_usage of T cells

File Name: Supplementary Data 6
Description: : List of ligand-receptor interaction pairs

File Name: Supplementary Data 7
Description: Antibody panels for multiplex fluorescent immunohistochemistry

File Name: Supplementary Data 8
Description: The malignant gene set and the nonmalignant gene set
