# Supplementary material for: scRNA-seq of gastric tumor shows complex intercellular interaction with an alternative T cell exhaustion trajectory
Source: Nat Commun. 2022 Aug 23;13:4943. doi: 10.1038/s41467-022-32627-z (PMC9399107; doi:10.1038/s41467-022-32627-z)
Supplement: Supplementary file 12 — Reporting Summary [file 41467_2022_32627_MOESM12_ESM.pdf]

nature portfolio

Corresponding author(s): Xun Lan, Yantao Tian

Last updated by author(s): May 1, 2022

Reporting Summary

Nature Portfolio wishes to improve the reproducibility of the work that we publish. This form provides structure for consistency and transparency in reporting. For further information on Nature Portfolio policies, see our Editorial Policies and the Editorial Policy Checklist.

Statistics

For all statistical analyses, confirm that the following items are present in the figure legend, table legend, main text, or Methods section.

n/a	Confirmed	
☐	☒	The exact sample size (*n*) for each experimental group/condition, given as a discrete number and unit of measurement
☐	☒	A statement on whether measurements were taken from distinct samples or whether the same sample was measured repeatedly
☐	☒	The statistical test(s) used AND whether they are one- or two-sided<br>*Only common tests should be described solely by name; describe more complex techniques in the Methods section.*
☐	☒	A description of all covariates tested
☐	☒	A description of any assumptions or corrections, such as tests of normality and adjustment for multiple comparisons
☐	☒	A full description of the statistical parameters including central tendency (e.g. means) or other basic estimates (e.g. regression coefficient) AND variation (e.g. standard deviation) or associated estimates of uncertainty (e.g. confidence intervals)
☐	☒	For null hypothesis testing, the test statistic (e.g. *F*, *t*, *r*) with confidence intervals, effect sizes, degrees of freedom and *P* value noted<br>*Give P values as exact values whenever suitable.*
☒	☐	For Bayesian analysis, information on the choice of priors and Markov chain Monte Carlo settings
☐	☒	For hierarchical and complex designs, identification of the appropriate level for tests and full reporting of outcomes
☐	☒	Estimates of effect sizes (e.g. Cohen's *d*, Pearson's *r*), indicating how they were calculated

Our web collection on statistics for biologists contains articles on many of the points above.

Software and code

Policy information about availability of computer code

Data collection	illumina HiSeq X Ten and NovaSeq 6000 System to generate the sequencing data.
Data analysis	Flow cytometry data was analyzed using FlowJo v10.<br>The stained slides were imaged and scanned using the Vectra Quantitative Pathology Imaging Systems and analyzed by Phenochart Image Analysis Software (Akoya Biosciences) version 1.0.12.<br>Transcriptome data was analyzed using BCL2fastq v2.20 (https://support.illumina.com/downloads/bcl2fastq-conversion-software-v2-20.html), Cell Ranger (https://support.10xgenomics.com/single-cell-gene-expression/software/release-notes/3-1, version3.1), R (2008 https://www.r-project.org/, version 3.5.1), R Studio (https://www.rstudio.com/, version 1.1.442), Python (https://www.python.org/,version 3.6.7), CellPhoneDB (https://github.com/Teichlab/cellphonedb, version 2.0.0), Cytoscape (https://cytoscape.org/, version 3.6.1), Seurat (https://github.com/satijalab/seurat/, version 3.1.0), InferCNV (https://github.com/broadinstitute/infercnv/releases/tag/InferCNV-v0.99.0, version 0.99.0), ggplot2 (https://ggplot2.tidyverse.org/), GSVA (https://github.com/rcastelo/GSVA, version1.30.0), pySCENIC (https://github.com/aertslab/SCENIC, version 0.11.0), DropEst (https://github.com/darneson/dropEst-1, version 0.8.6), GEPIA2 (http://gepia2.cancer-pku.cn/), Scanpy (https://github.com/theislab/scanpy, version 1.4.5; containing Leiden algorithm), scvelo(https://github.com/theislab/scvelo, version 0.1.25), Salmon (https://github.com/COMBINE-lab/salmon, ver. 1.6), Strelka2 (https://github.com/Illumina/strelka, ver. 2.9), VarTrix (https://github.com/10XGenomics/vartrix, ver. 1.1.22), MuSiC (https://xuranw.github.io/MuSiC/articles/MuSiC.html, ver. 0.2.0), cnvkit (https://github.com/etal/cnvkit,Version 0.9.9). In-house scripts are available at https://github.com/Lan-lab/sc-GC.

For manuscripts utilizing custom algorithms or software that are central to the research but not yet described in published literature, software must be made available to editors and reviewers. We strongly encourage code deposition in a community repository (e.g. GitHub). See the Nature Portfolio guidelines for submitting code & software for further information.

Data

Policy information about availability of data

All manuscripts must include a data availability statement. This statement should provide the following information, where applicable:

- Accession codes, unique identifiers, or web links for publicly available datasets
- A description of any restrictions on data availability
- For clinical datasets or third party data, please ensure that the statement adheres to our policy

The raw sequence data reported in this paper have been deposited in the Genome Sequence Archive in BIG Data Center, Beijing Institute of Genomics (BIG), Chinese Academy of Sciences. The raw sequence data are accessible at the following address (Access numbers: HRA000704): https://ngdc.cncb.ac.cn/gsa-human/browse/HRA000704. The processed expression matrices and cell annotations have been deposited into Open Archive for Miscellaneous Data (OMIX) database with accession ID: OMIX001073 (https://ngdc.cncb.ac.cn/omix/preview/UIeSVabl).

Field-specific reporting

Please select the one below that is the best fit for your research. If you are not sure, read the appropriate sections before making your selection.

☒ Life sciences ☐ Behavioural & social sciences ☐ Ecological, evolutionary & environmental sciences

For a reference copy of the document with all sections, see nature.com/documents/nr-reporting-summary-flat.pdf

Life sciences study design

All studies must disclose on these points even when the disclosure is negative.

Sample size	Ten stomach tumor tissues, eight paired adjacent paratumor tissues and four eripheral blood were profiled at single cell level. Number of cells per sample for single cell RNA sequencing was determined based on targeting parameters specified by 10x genomicsprotocols. The immunohistochemistry and multiplexed immunofluorescence staining assay were performed on six paraffin-embedded tumor and paratumor tissues.
Data exclusions	All criteria for data exclusion were pre-established. Firstly, Cells with less than 400 UMI counts, less than 200 genes, or greater than 30% of mitochondrial RNA counts were filtered. Secondly, cells expressing contradictory markers of known different cell types were removed as potential doublets. For T cells analysis, the filter threshold of mitochondrial RNA counts was altered to 20%. What's more, T cells with greater than 2 TRB or 2 TRA sequences were removed as potential doublets.
Replication	For GC03-tumor and GC10-paratumor tissues, two technical replicates of scRNA-seq library were processed to evaluate technical stability of 10x Genomics. The results about cancer-associated fibroblasts and macrophages represent the mean ± SD. from at least three independent experiments. All the replication were successful.
Randomization	The patients with stomach carcinoma were recruited randomly in this study. The paraffin-embedded tumor and paratumor tissues were randomly chosed for downstream immunohistochemistry and multiplexed immunofluorescence staining assay.
Blinding	The investigators were blinded to patient identity. Tumour biopsy was collected and sent for downstream procedures with coded Sample_ ID. Bacause this study is intended to investigate differenced between tumors and paratumors, the investigators were not blinded during single cell RNA-seq data analysis, cancer-associated fibroblasts and macrophages experiments, and staining assay.

Behavioural & social sciences study design

All studies must disclose on these points even when the disclosure is negative.

Study description	*Briefly describe the study type including whether data are quantitative, qualitative, or mixed-methods (e.g. qualitative cross-sectional, quantitative experimental, mixed-methods case study).*
Research sample	*State the research sample (e.g. Harvard university undergraduates, villagers in rural India) and provide relevant demographic information (e.g. age, sex) and indicate whether the sample is representative. Provide a rationale for the study sample chosen. For studies involving existing datasets, please describe the dataset and source.*
Sampling strategy	*Describe the sampling procedure (e.g. random, snowball, stratified, convenience). Describe the statistical methods that were used to predetermine sample size OR if no sample-size calculation was performed, describe how sample sizes were chosen and provide a rationale for why these sample sizes are sufficient. For qualitative data, please indicate whether data saturation was considered, and what criteria were used to decide that no further sampling was needed.*
Data collection	*Provide details about the data collection procedure, including the instruments or devices used to record the data (e.g. pen and paper, computer, eye tracker, video or audio equipment) whether anyone was present besides the participant(s) and the researcher, and whether the researcher was blind to experimental condition and/or the study hypothesis during data collection.*

Timing	*Indicate the start and stop dates of data collection. If there is a gap between collection periods, state the dates for each sample cohort.*
Data exclusions	*If no data were excluded from the analyses, state so OR if data were excluded, provide the exact number of exclusions and the rationale behind them, indicating whether exclusion criteria were pre-established.*
Non-participation	*State how many participants dropped out/declined participation and the reason(s) given OR provide response rate OR state that no participants dropped out/declined participation.*
Randomization	*If participants were not allocated into experimental groups, state so OR describe how participants were allocated to groups, and if allocation was not random, describe how covariates were controlled.*

Ecological, evolutionary & environmental sciences study design

All studies must disclose on these points even when the disclosure is negative.

Study description	*Briefly describe the study. For quantitative data include treatment factors and interactions, design structure (e.g. factorial, nested, hierarchical), nature and number of experimental units and replicates.*
Research sample	*Describe the research sample (e.g. a group of tagged Passer domesticus, all Stenocereus thurberi within Organ Pipe Cactus National Monument), and provide a rationale for the sample choice. When relevant, describe the organism taxa, source, sex, age range and any manipulations. State what population the sample is meant to represent when applicable. For studies involving existing datasets, describe the data and its source.*
Sampling strategy	*Note the sampling procedure. Describe the statistical methods that were used to predetermine sample size OR if no sample-size calculation was performed, describe how sample sizes were chosen and provide a rationale for why these sample sizes are sufficient.*
Data collection	*Describe the data collection procedure, including who recorded the data and how.*
Timing and spatial scale	*Indicate the start and stop dates of data collection, noting the frequency and periodicity of sampling and providing a rationale for these choices. If there is a gap between collection periods, state the dates for each sample cohort. Specify the spatial scale from which the data are taken*
Data exclusions	*If no data were excluded from the analyses, state so OR if data were excluded, describe the exclusions and the rationale behind them, indicating whether exclusion criteria were pre-established.*
Reproducibility	*Describe the measures taken to verify the reproducibility of experimental findings. For each experiment, note whether any attempts to repeat the experiment failed OR state that all attempts to repeat the experiment were successful.*
Randomization	*Describe how samples/organisms/participants were allocated into groups. If allocation was not random, describe how covariates were controlled. If this is not relevant to your study, explain why.*
Blinding	*Describe the extent of blinding used during data acquisition and analysis. If blinding was not possible, describe why OR explain why blinding was not relevant to your study.*

Did the study involve field work? ☐ Yes ☐ No

Field work, collection and transport

Field conditions	*Describe the study conditions for field work, providing relevant parameters (e.g. temperature, rainfall).*
Location	*State the location of the sampling or experiment, providing relevant parameters (e.g. latitude and longitude, elevation, water depth).*
Access & import/export	*Describe the efforts you have made to access habitats and to collect and import/export your samples in a responsible manner and in compliance with local, national and international laws, noting any permits that were obtained (give the name of the issuing authority, the date of issue, and any identifying information).*
Disturbance	*Describe any disturbance caused by the study and how it was minimized.*

Reporting for specific materials, systems and methods

We require information from authors about some types of materials, experimental systems and methods used in many studies. Here, indicate whether each material, system or method listed is relevant to your study. If you are not sure if a list item applies to your research, read the appropriate section before selecting a response.

Materials & experimental systems

n/a	Involved in the study	
☐	☒	Antibodies
☐	☒	Eukaryotic cell lines
☒	☐	Palaeontology and archaeology
☒	☐	Animals and other organisms
☐	☒	Human research participants
☒	☐	Clinical data
☒	☐	Dual use research of concern

Methods

n/a	Involved in the study	
☒	☐	ChIP-seq
☐	☒	Flow cytometry
☒	☐	MRI-based neuroimaging

Antibodies

Antibodies used

All antibodies were commercially purchased and included: anti-human CD3 (Mouse; BD Bioscience; Cat#565100; clone: HIT3a), anti-human CD45 (Mouse; BD Bioscience; Cat# 564105; clone: HI30), Fixable Viability Dye eFluor™ 506 (eBioscience, 65-0866-18), anti-human CD4 (Rabbit; Abcam; Cat#ab133616; clone:), anti-human CD8 (Rabbit; Abcam; Cat# ab93278; clone: EP1150Y), anti-human IL17A polyclonal antibody (Rabbit; Abcam; Cat# ab79056), AF700 anti-human CD45 (Mouse; BioLegend; Cat# 304023; clone: HI30), PB anti-EPCAM (Mouse; BioLegend; Cat# 324217; clone: 9C4), PE anti-CD31 (Mouse; BioLegend; Cat#303105; clone: WM59) , FITC anti-human HLA-DR, DP, DQ (Mouse; BioLegend; Cat# 361705; clone: Tü39), Anti-human CD68 (Rabbit; Abcam; Cat# ab283654; clone: EPR23917-164), Anti-human APOE (Rabbit; Abcam; Cat# ab52607; clone: EP1374Y), Anti-human THBS1 (Rabbit; Abcam; Cat# ab267388; clone: EPR22927-54), Anti-human CD31 (Rabbit; Abcam; Cat# ab281583; clone: RM1006), Anti-human FAP (Rabbit; Abcam; Cat# ab207178; clone: EPR20021), Anti-human HLA-DR (Rabbit; Abcam; Cat# ab92511; clone: EPR3692), Anti-human PDGFRA (Rabbit; Abcam; Cat# ab203491; clone: EPR22059-270), Anti-human TIGIT (Rabbit; Cell Signaling Technology; Cat# 99567T; clone: E5Y1W), Anti-human NECTIN2 (Rabbit; Abcam; Cat# ab233384; clone: EPR21124),anti-human CDX2 (Rabbit; CST; Cat 12306S; clone: D11D10), anit-Flag tag (CST, Cat 14793S; clone: D6W5B) ,anti-TFEC (abcam, Cat: ab185226; clone: EPR14871), anti-NR1H3 (LXR alpha; abcam, Cat ab176323; clone: EPR6508(N)), anti-human BMP1 (abcam, Cat: ab205394; clone:), anti-human WNT5A (abcam, Cat: ab179824; clone: EPR12698)

Validation

:All the antibodies used in this study were commercial antibodies, with validation procedures described on the following sites of the manufacturers:
anti-human CD3 (Mouse; BD Bioscience; Cat# 565100)
https://www.bdbiosciences.com/us/reagents/research/antibodies-buffers/immunology-reagents/anti-human-antibodies/cell-surface-antigens/bb515-mouse-anti-human-cd3-hit3a/p/565100
anti-human CD45 (Mouse; BD Bioscience; Cat# 564105)
https://www.bdbiosciences.com/us/applications/research/stem-cell-research/cancer-research/human/percp-cy55-mouse-anti-human-cd45-hi30/p/564105
Fixable Viability Dye eFluor™ 506 (eBioscience, 65-0866-18)
https://www.thermofisher.com/order/catalog/product/65-0866-18?SID=srch-hj-65-0866-18#/65-0866-18?SID=srch-hj-65-0866-18
anti-human CD4 (Rabbit; Abcam; Cat# ab133616)
https://www.abcam.com/cd4-antibody-epr6855-ab133616.html
anti-human CD8 (Rabbit; Abcam, Cat# ab93278)
https://www.abcam.com/cd8-alpha-antibody-ep1150y-ab93278.html
anti-human IL17A (Rabbit; Abcam; Cat# ab79056)
https://www.abcam.com/il-17a-antibody-ab79056.html
AF700 anti-human CD45 (Mouse; BioLegend; Cat# 304023)
https://www.biolegend.com/en-us/search-results/alexa-fluor-700-anti-human-cd45-antibody-3401
PB anti-EPCAM (Mouse; BioLegend; Cat# 324217)
https://www.biolegend.com/en-us/products/pacific-blue-anti-human-cd326-epcam-antibody-6931
PE anti-CD31 (Mouse; BioLegend; Cat#303105)
https://www.biolegend.com/en-us/products/pe-anti-human-cd31-antibody-882
FITC anti-human HLA-DR, DP, DQ (Mouse; BioLegend; Cat# 361705)
https://www.biolegend.com/en-us/products/fitc-anti-human-hla-dr-dp-dq-antibody-9376
Anti-human CD68 (Rabbit; Abcam; Cat# ab283654)
https://www.abcam.com/cd68-antibody-epr23917-164-ab283654.html
Anti-human APOE (Rabbit; Abcam; Cat# ab52607)
https://www.abcam.com/apolipoprotein-e-antibody-ep1374y-ab52607.html
Anti-human THBS1 (Rabbit; Abcam; Cat# ab267388)
https://www.abcam.com/thrombospondin-1-antibody-epr22927-54-ab267388.html
Anti-human CD31 (Rabbit; Abcam; Cat# ab281583)
https://www.abcam.com/cd31-antibody-rm1006-ab281583.html
Anti-human FAP (Rabbit; Abcam; Cat# ab207178)
https://www.abcam.com/fibroblast-activation-protein-alpha-antibody-epr20021-ab207178.html
Anti-human HLA-DR (Rabbit; Abcam; Cat# ab92511)
https://www.abcam.com/hla-dr-antibody-epr3692-ab92511.html
Anti-human PDGFRA (Rabbit; Abcam; Cat# ab203491)
https://www.abcam.com/pdgfr-alpha-antibody-epr22059-270-ab203491.html
Anti-human TIGIT (Rabbit; Cell Signaling Technology; Cat# 99567T)

https://www.cellsignal.com/products/primary-antibodies/tigit-e5y1w-xp-rabbit-mab/99567?site-search-type=Products&N=4294956287&Ntt=99567t&fromPage=plp&_requestid=851342
Anti-human NECTIN2 (Rabbit; Abcam; Cat: ab233384)
https://www.abcam.com/nectin-2-antibody-epr21124-ab233384.html
anti-human CDX2 (Rabbit; CST; Cat: 12306S)
https://www.cellsignal.cn/products/primary-antibodies/cdx2-d11d10-rabbit-mab/12306?site-search-type=Products&N=4294956287&Ntt=cdx2&fromPage=plp
anit-Flag tag (CST, Cat 14793S)
https://www.cellsignal.cn/products/primary-antibodies/dykddddk-tag-d6w5b-rabbit-mab-binds-to-same-epitope-as-sigma-s-anti-flag-m2-antibody/14793?site-search-type=Products&N=4294956287&Ntt=flag&fromPage=plp
anti-human TFEC (abcam, Cat: ab185226)
https://www.abcam.cn/tfec-antibody-epr14871-ab185226.html
anti-human NR1H3 (abcam, Cat: ab176323)
https://www.abcam.cn/lxr-alpha-antibody-epr6508n-ab176323.html
anti-human BMP1 (abcam, Cat: ab205394)
https://www.abcam.cn/bmp1pcp-antibody-ab205394.html
anti-human WNT5A (abcam, Cat: ab179824)
https://www.abcam.cn/wnt5a-antibody-epr12698-c-terminal-ab179824.html

Eukaryotic cell lines

Policy information about cell lines

Cell line source(s)

THP-1, SNU-16, KATO III, MKN-45, SNU-1, AGS, SGC-7901, HGC-27, MKN-28 and MKN-7 cell lines were from the American Type Culture Collection (Manassas, VA). THP-1-TFEC/NR1H3, HGC-27/SGC-7901/MKN-28-CDX2/HOXA13/NR1I2 overexpressed cell lines were established by lentivirus infection of the corresponding plasmid, which all were confirmed by western blot.

Authentication

THP-1, SNU-16, KATO III, MKN-45, SNU-1, AGS, SGC-7901, HGC-27, MKN-28 and MKN-7 cell lines were authenticated by ATCC by using STR profiling.

Mycoplasma contamination

Mycoplasma contamination was negative based on the PCR results with ATCC Universal Mycoplasma Detection Kit (cat. no. 30-1012K)

Commonly misidentified lines
(See ICLAC register)

No

Palaeontology and Archaeology

Specimen provenance

Provide provenance information for specimens and describe permits that were obtained for the work (including the name of the issuing authority, the date of issue, and any identifying information). Permits should encompass collection and, where applicable, export.

Specimen deposition

Indicate where the specimens have been deposited to permit free access by other researchers.

Dating methods

If new dates are provided, describe how they were obtained (e.g. collection, storage, sample pretreatment and measurement), where they were obtained (i.e. lab name), the calibration program and the protocol for quality assurance OR state that no new dates are provided.

☐ Tick this box to confirm that the raw and calibrated dates are available in the paper or in Supplementary Information.

Ethics oversight

Identify the organization(s) that approved or provided guidance on the study protocol, OR state that no ethical approval or guidance was required and explain why not.

Note that full information on the approval of the study protocol must also be provided in the manuscript.

Animals and other organisms

Policy information about studies involving animals; ARRIVE guidelines recommended for reporting animal research

Laboratory animals

For laboratory animals, report species, strain, sex and age OR state that the study did not involve laboratory animals.

Wild animals

Provide details on animals observed in or captured in the field; report species, sex and age where possible. Describe how animals were caught and transported and what happened to captive animals after the study (if killed, explain why and describe method; if released, say where and when) OR state that the study did not involve wild animals.

Field-collected samples

For laboratory work with field-collected samples, describe all relevant parameters such as housing, maintenance, temperature, photoperiod and end-of-experiment protocol OR state that the study did not involve samples collected from the field.

Ethics oversight

Identify the organization(s) that approved or provided guidance on the study protocol, OR state that no ethical approval or guidance was required and explain why not.

Note that full information on the approval of the study protocol must also be provided in the manuscript.

Human research participants

Policy information about studies involving human research participants

Population characteristics	Thirty patients who were pathologically diagnosed with stomach carcinoma were enrolled in this study. None of the patients had received prior treatment. Detailed information can be found in the Clinical sample collection section of Methods and Supplementary Data 1.
Recruitment	All donors are recruited and managed by Cancer Hospital, Chinese Academy of Medical Sciences, avoiding the selection of poorly clinically characterized volunteers. Thirty gastric cancer patients who were pathologically diagnosed with GC were enrolled in this project, and they received none chemotherapy, radiation, or drug treatment before tumor resection. Due to limited samples used in this study, thus future work based on larger cohorts of gastric cancer patients are needed to generalize our findings.
Ethics oversight	This study complies with all relevant ethical regulations and was approved by the medical ethics committee of the Institutional Review Board of the Cancer Hospital Chinese Academy of Medical Sciences. THe written informed consent was provided by all participants.

Note that full information on the approval of the study protocol must also be provided in the manuscript.

Clinical data

Policy information about clinical studies

All manuscripts should comply with the ICMJEguidelines for publication of clinical research and a completedCONSORT checklist must be included with all submissions.

Clinical trial registration	*Provide the trial registration number from ClinicalTrials.gov or an equivalent agency.*
Study protocol	*Note where the full trial protocol can be accessed OR if not available, explain why.*
Data collection	*Describe the settings and locales of data collection, noting the time periods of recruitment and data collection.*
Outcomes	*Describe how you pre-defined primary and secondary outcome measures and how you assessed these measures.*

Dual use research of concern

Policy information about dual use research of concern

Hazards

Could the accidental, deliberate or reckless misuse of agents or technologies generated in the work, or the application of information presented in the manuscript, pose a threat to:

No	Yes	
☐	☐	Public health
☐	☐	National security
☐	☐	Crops and/or livestock
☐	☐	Ecosystems
☐	☐	Any other significant area

Experiments of concern

Does the work involve any of these experiments of concern:

No	Yes	
☐	☐	Demonstrate how to render a vaccine ineffective
☐	☐	Confer resistance to therapeutically useful antibiotics or antiviral agents
☐	☐	Enhance the virulence of a pathogen or render a nonpathogen virulent
☐	☐	Increase transmissibility of a pathogen
☐	☐	Alter the host range of a pathogen
☐	☐	Enable evasion of diagnostic/detection modalities
☐	☐	Enable the weaponization of a biological agent or toxin
☐	☐	Any other potentially harmful combination of experiments and agents

ChIP-seq

Data deposition

☐ Confirm that both raw and final processed data have been deposited in a public database such as GEO.

☐ Confirm that you have deposited or provided access to graph files (e.g. BED files) for the called peaks.

Data access links<br>*May remain private before publication.*	*For "Initial submission" or "Revised version" documents, provide reviewer access links. For your "Final submission" document, provide a link to the deposited data.*
Files in database submission	*Provide a list of all files available in the database submission.*
Genome browser session<br>(e.g. UCSC)	*Provide a link to an anonymized genome browser session for "Initial submission" and "Revised version" documents only, to enable peer review. Write "no longer applicable" for "Final submission" documents.*

Methodology

Replicates	*Describe the experimental replicates, specifying number, type and replicate agreement.*
Sequencing depth	*Describe the sequencing depth for each experiment, providing the total number of reads, uniquely mapped reads, length of reads and whether they were paired- or single-end.*
Antibodies	*Describe the antibodies used for the ChIP-seq experiments; as applicable, provide supplier name, catalog number, clone name, and lot number.*
Peak calling parameters	*Specify the command line program and parameters used for read mapping and peak calling, including the ChIP, control and index files used.*
Data quality	*Describe the methods used to ensure data quality in full detail, including how many peaks are at FDR 5% and above 5-fold enrichment.*
Software	*Describe the software used to collect and analyze the ChIP-seq data. For custom code that has been deposited into a community repository, provide accession details.*

Flow Cytometry

Plots

Confirm that:

☒ The axis labels state the marker and fluorochrome used (e.g. CD4-FITC).

☒ The axis scales are clearly visible. Include numbers along axes only for bottom left plot of group (a 'group' is an analysis of identical markers).

☒ All plots are contour plots with outliers or pseudocolor plots.

☒ A numerical value for number of cells or percentage (with statistics) is provided.

Methodology

Sample preparation	Briefly, fresh tissue samples were cut into small slices and enzymatically digested in the 10 ml RPMI-1640 medium containing 10% fetal bovine serum (FBS; GIBCO, Cat: 16000044), 1mg/ml Collagenase type II (Gibco, Cat: 17101015), 1mg/ml Collagenase type IV (Gibco, Cat: 17104019), 2mg/ml Dispase II (Roche, Cat: 4942078001), 1mg/mL DNase I (Roche, Cat:10104159001), at 37℃ with 150 rpm rotation for 30 minutes. Following digestion, digested tissue pieces were passed through a 70-µm filter. The suspended cells were spun down at 400 g at 4℃ for 5 min and resuspended in ACK lysis buffer for

3 minutes on ice to remove red blood cells. After washing twice with 1x PBS (GIBCO, Cat: C10010500BT), the cell pellets were resuspended in sorting buffer (PBS containing 2% FBS). Peripheral blood mononuclear cells (PBMCs) were isolated using HISTOPAQUE-1077 (Sigma-Aldrich, Cat: 10771) solution according to the manufacturer instructions. After red blood cells were removed via the same procedure described above, PBMCs were also resuspended in the sorting buffer. All samples were stained for sorting at 1x106 cells per ml for 20 min on ice with Fixable Viability Dye eFluor™ 506 (eBioscience, Cat: 65-0866-18; 1:1000) for live-dead discrimination. For patients GC02 and GC08, tumors were stained with PerCP-Cy5.5 Mouse Anti-Human CD45 (BD Bioscience, Cat: 564105; 1:200) and PE Mouse Anti-Human CD3 (BD Bioscience, Cat: 555340; 1:200) antibodies to additionally isolate and enrich tumor-infiltrating T cells. Fluorescence-activated cell sorting (FACS) was performed on a FACSAria III instrument (BD Biosciences).
For comparing the proportion of MHC class II positive endothelium in tumor versus para-tumor tissue, single-cell suspensions of paired tumor and paratumor tissues from another nine patients were stained with Fixable Viability Dye eFluor™ 506 (1:1000), AF700 mouse anti-human CD45 (BioLegend, Cat: 304023; 1:200), PB mouse anti-EPCAM (BioLegend, Cat: 324217; 1:200), PE mouse anti-CD31 (BioLegend, Cat: 303105; 1:200) and FITC mouse anti-HLA- HLA-DR, DP, DQ (BioLegend, Cat: 361705; 1:200) antibodies.

Instrument: BD FACSAria III

Software: FlowJo v10

Cell population abundance: Up to 10^6 single viable cells were sorted from each PBMC, paratumor, or tumour sample

Gating strategy: Information available on supplementary Fig. 1a and supplementary Fig. 3c, and Methods sections. Live cells were firstly gated by FSC-A and SSC-A to exclude the debris, followed by FSC-H to gate single cells. Dead cells were exclued by using viability dye. CD3+&CD45+ cells were used to identify T cells; CD45-&EPCAM-&CD31+ were used to idnetify endothelial cells.

☒ Tick this box to confirm that a figure exemplifying the gating strategy is provided in the Supplementary Information.

Magnetic resonance imaging

Experimental design

Design type: *Indicate task or resting state; event-related or block design.*

Design specifications: *Specify the number of blocks, trials or experimental units per session and/or subject, and specify the length of each trial or block (if trials are blocked) and interval between trials.*

Behavioral performance measures: *State number and/or type of variables recorded (e.g. correct button press, response time) and what statistics were used to establish that the subjects were performing the task as expected (e.g. mean, range, and/or standard deviation across subjects).*

Acquisition

Imaging type(s): *Specify: functional, structural, diffusion, perfusion.*

Field strength: *Specify in Tesla*

Sequence & imaging parameters: *Specify the pulse sequence type (gradient echo, spin echo, etc.), imaging type (EPI, spiral, etc.), field of view, matrix size, slice thickness, orientation and TE/TR/flip angle.*

Area of acquisition: *State whether a whole brain scan was used OR define the area of acquisition, describing how the region was determined.*

Diffusion MRI ☐ Used ☐ Not used

Preprocessing

Preprocessing software: *Provide detail on software version and revision number and on specific parameters (model/functions, brain extraction, segmentation, smoothing kernel size, etc.).*

Normalization: *If data were normalized/standardized, describe the approach(es): specify linear or non-linear and define image types used for transformation OR indicate that data were not normalized and explain rationale for lack of normalization.*

Normalization template: *Describe the template used for normalization/transformation, specifying subject space or group standardized space (e.g. original Talairach, MNI305, ICBM152) OR indicate that the data were not normalized.*

Noise and artifact removal: *Describe your procedure(s) for artifact and structured noise removal, specifying motion parameters, tissue signals and physiological signals (heart rate, respiration).*

Volume censoring: *Define your software and/or method and criteria for volume censoring, and state the extent of such censoring.*

Statistical modeling & inference

Model type and settings | *Specify type (mass univariate, multivariate, RSA, predictive, etc.) and describe essential details of the model at the first and second levels (e.g. fixed, random or mixed effects; drift or auto-correlation).*

Effect(s) tested | *Define precise effect in terms of the task or stimulus conditions instead of psychological concepts and indicate whether ANOVA or factorial designs were used.*

Specify type of analysis: ☐ Whole brain ☐ ROI-based ☐ Both

Statistic type for inference (See Eklund et al. 2016) | *Specify voxel-wise or cluster-wise and report all relevant parameters for cluster-wise methods.*

Correction | *Describe the type of correction and how it is obtained for multiple comparisons (e.g. FWE, FDR, permutation or Monte Carlo).*

Models & analysis

n/a	Involved in the study
☐	☐ Functional and/or effective connectivity
☐	☐ Graph analysis
☐	☐ Multivariate modeling or predictive analysis

Functional and/or effective connectivity | *Report the measures of dependence used and the model details (e.g. Pearson correlation, partial correlation, mutual information).*

Graph analysis | *Report the dependent variable and connectivity measure, specifying weighted graph or binarized graph, subject- or group-level, and the global and/or node summaries used (e.g. clustering coefficient, efficiency, etc.).*

Multivariate modeling and predictive analysis | *Specify independent variables, features extraction and dimension reduction, model, training and evaluation metrics.*
